# Mathematical analysis of left ventricular elastance with respect to afterload change during ejection phase

**DOI:** 10.1371/journal.pcbi.1011974

**Published:** 2024-04-18

**Authors:** Shiro Kato, Yukiko Himeno, Akira Amano

**Affiliations:** Department of Bioinformatics, Ritsumeikan University, Kusatsu, Shiga, Japan; Stanford University, UNITED STATES

## Abstract

Since the left ventricle (LV) has pressure (*P*_*lv*_) and volume (*V*_*lv*_), we can define LV elastance from the ratio between *P*_*lv*_ and *V*_*lv*_, termed as “instantaneous elastance.” On the other hand, end-systolic elastance (*E*_*max*_) is known to be a good index of LV contractility, which is measured by the slope of several end-systolic *P*_*lv*_—*V*_*lv*_ points obtained by using different loads. The word *E*_*max*_ originates from the assumption that LV elastance increases during the ejection phase and attains its maximum at the end-systole. From this concept, we can define another elastance determined by the slope of isochronous *P*_*lv*_—*V*_*lv*_ points, that is *P*_*lv*_—*V*_*lv*_ points at a certain time after the ejection onset time by using different loads. We refer to this elastance as “load-dependent elastance.”

To reveal the relation between these two elastances, we used a hemodynamic model that included a detailed ventricular myocyte contraction model. From the simulation results, we found that the isochronous *P*_*lv*_—*V*_*lv*_ points lay in one line and that the line slope corresponding to the load-dependent elastance slightly decreased during the ejection phase, which is quite different from the instantaneous elastance.

Subsequently, we analyzed the mechanism determining these elastances from the model equations. We found that instantaneous elastance is directly related to contraction force generated by the ventricular myocyte, but the load-dependent elastance is determined by two factors: one is the transient characteristics of the cardiac cell, i.e., the velocity–dependent force drops characteristics in instantaneous shortening. The other is the force–velocity relation of the cardiac cell. We also found that the linear isochronous pressure–volume relation is based on the approximately linear relation between the time derivative of the cellular contraction force and the cellular shortening velocity that results from the combined characteristics of LV and aortic compliances.

## Author summary

Characteristic that the end-systolic pressure volume relation (ESPVR) of left ventricle becomes linear is one of the well known property of the heart, and its slope is considered as a good index of the heart contractility. However, the reason of the linearity is still not clarified. Recent biophysical model can reproduce the linear ESPVR, and since the model is written in the mathematical equation, the basis of the ESPVR linearity can be analytically analyzed.

## Introduction

The primary function of the heart is to transport arterial blood to the various organs and venous blood to the lungs by the ventricular myocyte contractile force. Since the pump function is impaired in the pathological conditions, quantitative indices which represents the heart contractility was interested by clinical cardiologists which may become an early indication of myocardial disease [1]. The pump function of the heart has been measured by many indices such as cardiac output, stroke volume, peak systolic pressure, and rate of rise of left ventricular pressure. However, since there are muscle cell characteristics that the contraction force increases with its length (force length relation), and decreases with its velocity (force velocity relation), measurements of these indices are affected by the preload which determines the cellular length at end-diastole, and afterload which determines the cellular contraction velocity during ejection phase [2]. Thus a single universal quantitative description of the cardiac pump function was investigated in 1970s, and Suga and Sagawa has proposed *E*_*max*_ as an index of cardiac pump function which is not affected by the cardiac load conditions.

*E*_*max*_ is a well-known index of cardiac contractility, which is the slope of the end-systolic pressure-volume relation (ESPVR), where ESPVR is obtained from the end-systolic points of several pressure-volume loops (PV loops) by using different preloads or afterloads on the left ventricle (LV) [3, 4]. The word *E*_*max*_ represents the maximum elastance of LV, and the concept that it represents the contractility of LV is based on the assumption that the LV elastance increases during the ejection phase and reaches its maximum value at the end-systole. *E*_*max*_ is considered a good index of LV contractility as it was considered to be load independent.

There are many researches on *E*_*max*_. For example, several studies have found that *E*_*max*_ increases with adrenergic stimulation [4–8], and decreases in ailing conditions such as heart failure [9–11].

However, in several studies, *E*_*max*_ was found to be afterload-dependent [12] and ESPVR to not be linear but convex [13, 14]. Moreover, it was found that ESPVR’s volume intercept will move with load [15, 52]. These points are briefly summarized in [16].

*E*_*max*_ is experimentally obtained by gradual preload reduction [17]. Since this approach is not easy to apply in the clinical situation, methods to estimate *E*_*max*_ have been suggested by using a single PV loop [11]. However, there is still no good method to estimate *E*_*max*_, limiting its applicability in clinical practice.

These limitations suggest that the theoretical basis of the *E*_*max*_ is still not clear and requires further investigation.

In the related reports, *E*_*max*_ are measured by the slope of pressure and volume relation at end-systole. By generalizing this elastance to the arbitrary time point during ejection phase, we can think of the elastance as the slope of pressure and volume relation at given time, and this elastance is represented as “load-dependent elastance” (*E*_*load*_) in this paper, where several pressure and volume points with different LV loads at a given time are necessary to determine it.

On the other hand, as we can consider LV as a compartment with pressure and volume, from the amount of the pressure change caused by the instantaneous volume change, the physical elastance of LV can be defined by the ratio between the pressure and volume change. This elastance can be recognized as a physical elastance property at a given time. Thus, in this paper this elastance is represented as “instantaneous elastance” (*E*_*inst*_).

Historically, Templeton et al. measured the ratio between the pressure and the volume change by applying the 22 Hz sinusoidal volume change to canine LV [18]. From our definition, we can consider this ratio to be close to *E*_*inst*_. Additionally, they reported that this value was linearly related to pressure throughout the cardiac cycle. However, the temporal change in the ratio was not clearly reported.

Technically, it is very difficult to directly measure LV elastance. There are several reports on the measurement of cardiac tissue elastances [19–22]. Saeki et al. measured kitten’s papillary muscle stiffness by applying an oscillation of 0.1 to 60 Hz and reported that stiffness changes with the cardiac cycle, but the amplitude of the difference was not large [23].

Historically, instantaneous elastance and load–dependent elastance were conceptually considered to represent similar properties of LV. However, there are very few reports on the measurement of instantaneous elastance; thus, it is difficult to compare these two. We now have cellular contraction models based on molecular level findings capable of reproducing muscle kinetic properties. Therefore, in this paper we try to analyze the characteristics of instantaneous elastance and load–dependent elastance based on the molecular level muscle contraction model by using the hemodynamic model combined with the contraction model.

## Model and definitions

### Model structure

In this research, a simplified hemodynamic model proposed by our group [24, 25] was used for mathematical analysis. The model was constructed from a circulation model, a LV geometry model, and a muscle contraction model.

The variables used in the model equations are summarized in Table 1.

**Table 1:** Variables of the simplified circulation model.

Variables	units	meaning
*t*	[ms]	simulation time
*P*_*a*_	[mmHg]	aortic pressure
*V*_*a*_	[mL]	aortic volume
*P*_*lv*_	[mmHg]	LV pressure
*V*_*lv*_	[mL]	LV volume
*R*_*lv*_	[cm]	LV internal radius
*h*_*lv*_	[cm]	LV wall thickness
*L*	[*μ*m]	half-sarcomere length
*F*_*ext*_	[mN/mm^2^]	LV wall tension
*F*_*b*_	[mN/mm^2^]	cellular contraction force
*F*_*p*_	[mN/mm^2^]	passive elastic force
$TSCa_{3}^{*}$	[*μ*M]	troponin bound Ca^2+^ at power state
$TSCa_{3}^{∼}$	[*μ*M]	troponin bound Ca^2+^ at weak state
TS*	[*μ*M]	troponin at power state
*TS* ^∼^	[*μ*M]	troponin at weak state
*h*_*p*_	[*μ*m]	crossbridge length at power state
*h*_*w*_	[*μ*m]	crossbridge length at weak state
*X*_*p*_	[*μ*m]	inextensible portion of L at power state
*X*_*w*_	[*μ*m]	inextensible portion of L at weak state
*Q*_*rel*_	[*μ*M/ms]	Ca^2+^ release flux
*Q*_*pump*_	[*μ*M/ms]	Ca^2+^ uptake flux

### Circulation model

**Fig 1:**
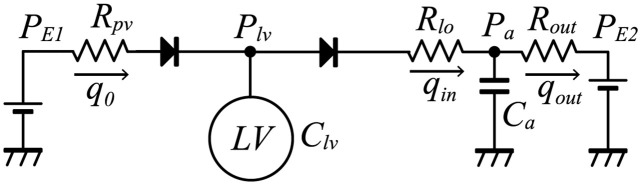
Circulation model.

Because our aim was to mathematically analyze the pressure and volume relation of the model, we used a simplified circulation model based on the Windkessel model (Fig 1). The parameters in the model were basically imported from the circulation model proposed by Heldt et al. [26] and Liang et al. [27].

The model included pulmonary venous pressure (*P*_*E*1_), LV pressure (*P*_*lv*_), aortic pressure (*P*_*a*_), and peripheral pressure (*P*_*E*2_) as pressure variables. For simplicity, we used constant values for *P*_*E*1_ and *P*_*E*2_. The model also included pulmonary venous resistance (*R*_*pv*_), aortic resistance (*R*_*lo*_), and peripheral resistance (*R*_*out*_) as vascular resistance parameters. The LV volume was denoted by *V*_*lv*_ and the aortic volume was denoted by *V*_*a*_. The flow between compartments were denoted as *q*_0_ for the LV incoming blood flow, *q*_*in*_ for the aortic blood flow, and *q*_*out*_ for the peripheral blood flow. The aortic compliance was denoted by *C*_*a*_, which has a relation with the aortic pressure and volume as follows.

$$\begin{array}{c} P_{a}=\frac{V_{a}}{C_{a}} \end{array}$$ (1)

As our aim was to reproduce the baseline hemodynamics, we did not include the baroreflex effect in the model. Moreover, we fixed the cycle length at 1000 [ms]. To evaluate the effect of changes in the afterload, we used several values for *R*_*out*_. The parameters used in the circulation model are shown in Table 2.

**Table 2:** Parameters of the circulation model.

*P*_*E*1_	*P*_*E*2_	*R*_*pv*_	*R*_*lo*_	*R*_*out*_
[mmHg]	[mmHg]	[mmHg ⋅ ms/mL]	[mmHg ⋅ ms/mL]	[mmHg ⋅ ms/mL]
10.0	15.0	27.0	2.0	1050—2050

*C*_*lv*_	*C*_*a*_
[mL/mmHg]	[mL/mmHg]
variable	1.5

### LV geometric model

As our analysis was based on the molecular-level contraction model, a LV geometry model was necessary to relate LV pressure and volume with cellular level contraction force and sarcomere length. We used a measurement-based fitting function that relates 1) the LV internal radius (*R*_*lv*_) to *V*_*lv*_, and 2) *R*_*lv*_ with half-sarcomere length (*L*), respectively. Also, Laplace’s law [28] was used to relate the LV wall tension (*F*_*ext*_) with LV pressure (*P*_*lv*_).

**Fig 2:**
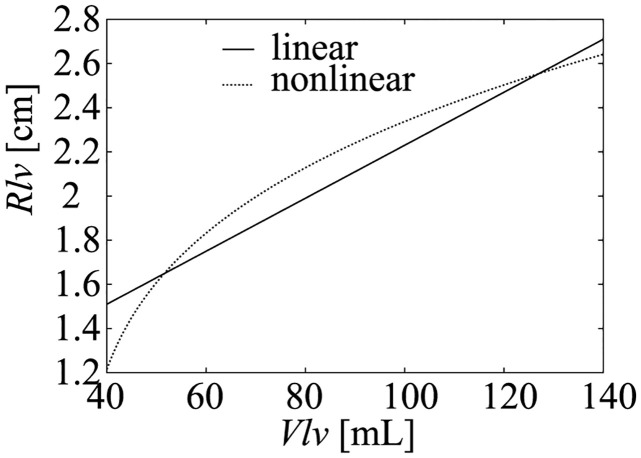
Relation between *R*_*lv*_ and *V*_*lv*_ obtained from the data reported by Rodriguez et al. [31] and Sabbah et al. [32] (non-linear), and linearized function used in the model (linear).

**Fig 3:**
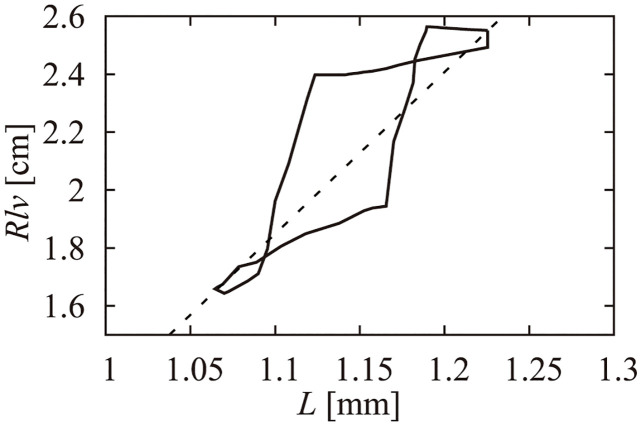
Relation between LV radius *R*_*lv*_ and half sarcomere length *L* obtained from data by Rodriguez [31] and Sabbah [32], and approximated linear relation (dotted line).

- Relation between the LV volume and internal radius
  Utaki et al. [24, 25] defined the equation for the relation between *R*_*lv*_ and *V*_*lv*_ using the following reported data. Corsi et al. [29] measured the time course of the human LV volume, and Sutton et al. [30] measured the time course of the human LV internal radius. Combining these data, a non-linear relation between the LV volume and the internal radius was obtained. However, as the resolution of these data was insufficient, they used the time course of the canine LV volume reported by Rodriguez et al. [31] and the time course of the canine LV internal diameter reported by Sabbah et al. [32] to draw a non-linear relation between the LV volume and internal radius. Given that these are canine data, scaling to human data was performed, and the non-linear equation between the LV volume and internal radius was obtained (Fig 2 nonlinear curve). In this study, to simplify the mathematical analysis, we approximated the non-linear equation between the LV volume and internal radius into the linear equation as follows (Fig 2 linear line).
  $$\begin{array}{c} \begin{array}{c} R_{lv}=K_{R}V_{lv}+K_{V} \end{array} \end{array}$$ (2)
  Here, *K*_*R*_ and *K*_*V*_ are constants adjusted to the physiological relation between *R*_*lv*_ and *V*_*lv*_ during end-diastole to end-systole.
- Relation between the LV myocardial sarcomere length and internal radius
  Utaki et al. [24, 25] used the following reported data to define the relation between *R*_*lv*_ and *L*. Rodriguez et al. [31] measured the time course of canine LV sarcomere length, and Sabbah et al. measured the time course of the canine LV internal diameter [32]. By combining these two, a non-linear relation between the LV volume and sarcomere length was obtained (Fig 3). Also it was assumed that the characteristics were basically similar with canines and humans, thus the scaling factor to the relation was introduced. In this study, we used the same equation shown in Eq (3) that Utaki et al. [24] used (Fig 3 dotted line).
$$\begin{array}{c} L=K_{L}R_{lv}+L_{b} \end{array}$$ (3)
  Here, *K*_*L*_ and *L*_*b*_ are constants.
- Simplified LV wall thickness equation
  LV wall thickness is known to become maximal at the end-systole and minimal at the end-diastole. In a recent report, not only the LV volume but also the LV twist angle was found to be related to the wall thickness [33]. Thus, wall thickness is not always proportional to the LV volume [29, 30], and the quantitative mechanism of wall thickness remains unclear. Utaki et al. [24] assumed that wall thickness is linearly related to the cellular contraction force. However, in this study, to simplify the mathematical analysis, we used constant wall thickness during ejection phase.
  $$\begin{array}{c} \begin{array}{c} h_{lv}=h_{lvED} \end{array} \end{array}$$ (4)
  Here, *h*_*lv*_ [cm] denotes the LV wall thickness. Utaki et al. [24] assumed *h*_*lv*_ = 1.00 [cm] at the end-diastole and *h*_*lv*_ = 1.70 [cm] at the end-systole; thus, we used *h*_*lvED*_ = 1.35 [cm] as the constant wall thickness in our analysis.
- Laplace’s law
  As Laplace’s law represents well the relation between *P*_*lv*_, *h*_*lv*_, *F*_*ext*_, and *R*_*lv*_ [34], we used this relation in our model.
  $$\begin{array}{c} P_{lv}=\frac{2K_{u}F_{ext}h_{lv}}{R_{lv}} \end{array}$$ (5)
  Here, *F*_*ext*_ [mN/mm^2^] denotes the LV wall tension. The calculation method of *F*_*ext*_ is explained in section Muscle cell contraction model. *K*_*u*_ is a parameter that converts units from [mN/mm^2^] to [mmHg].

To reproduce the physiological hemodynamic parameters, we used the model parameter values shown in Table 3.

**Table 3:** Parameters of the LV geometry model.

*K*_*R*_ [cm/mL]	*K*_*V*_ [cm]	*K*_*L*_ [*μ*m/cm]	*L*_*b*_ [*μ*m]	*K*_*S*_	*K*_*u*_ [mmHg/mN/mm^2^]	*h*_*lvED*_ [cm]
0.012	1.03	0.163	0.682	7.24	7.50064	1.35

### Muscle cell contraction model

**Fig 4:**
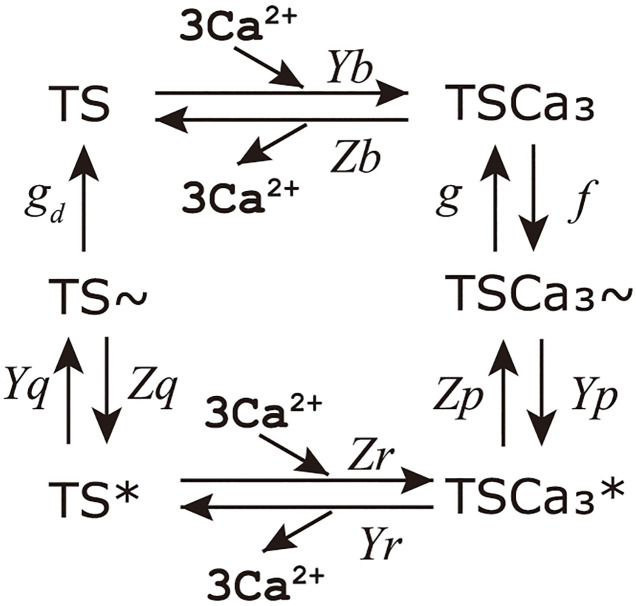
Chemical state transition and rate constants of NL08 model.

**Fig 5:**
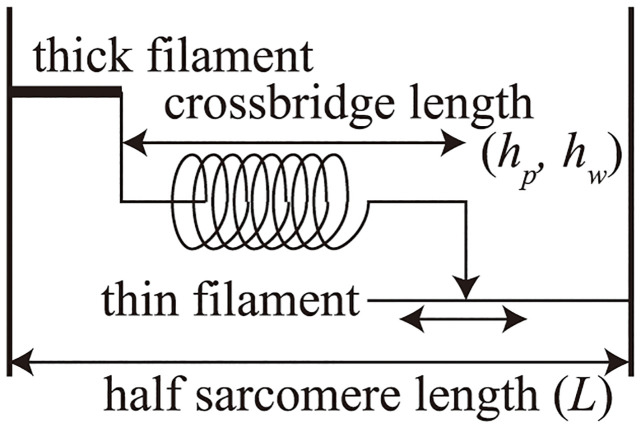
Mechanical model of NL08 model. Contraction force is determined by the spring rate determined by the chemical state model multiplied by the crossbridge length (*h*_*p*_, *h*_*w*_). Note that the crossbridge consists of weak (w) and power (p) states.

In this study, we used a muscle cell contraction model proposed by Negroni and Lascano (NL08 model) [35]. NL08 model consists of chemical state transition model shown in Fig 4 and mechanical model which represents elongating spring which has sliding connection point shown in Fig 5. In the model, the cellular contraction force (*F*_*b*_ [mN/mm^2^]) is calculated by multiplying the probability of the power generation states of the troponin system and crossbridge length (*h*_*p*_ [*μ*m], *h*_*w*_ [*μ*m]).

$$\begin{array}{ccc} F_{b} & = & A_{w}\left(\left[TSCa_{3}^{∼}\right]+\left[TS^{∼}\right]\right)h_{w}+A_{p}\left(\left[TSCa_{3}^{*}\right]+\left[TS^{*}\right]\right)h_{p} \end{array}$$ (6)

$$\begin{array}{c} h_{w}=L-X_{w} \end{array}$$ (7)

$$\begin{array}{c} h_{p}=L-X_{p} \end{array}$$ (8)

$$\begin{array}{c} \frac{dX_{p}}{dt}=B\left(h_{p}-h_{pr}\right) \end{array}$$ (9)

$$\begin{array}{c} \frac{dX_{w}}{dt}=B\left(h_{w}-h_{wr}\right) \end{array}$$ (10)

*A*_*w*_ and *A*_*p*_ represent the spring rate of crossbridges in the weak (w) and power (p) states that generate *F*_*b*_. [$TSCa_{3}^{∼}$] [*μ*M], [*TS*^∼^] [*μ*M], [$TSCa_{3}^{*}$] [*μ*M] and [TS*] [*μ*M] represent the concentrations of the troponin system forming the crossbridges in the weak (∼) and the power (*) states. As shown in the following equations, changes in these states are calculated by the rate constants shown in Fig 4, where the *Ca*^2+^ bound troponin system state TSCa_3_ increases from free troponin TS with increase in *Ca*^2+^ concentration, and crossbridge forming state TSCa_3^∼^_ increases with rate constant *f*.

$$\begin{array}{c} \frac{d[TSCa_{3}]}{dt}=Y_{b}\left[TS\right]{\left[Ca^{2+}\right]}^{3}-Z_{b}\left[TSCa_{3}\right]+g\left[TSCa_{3}^{∼}\right]-f\left[TSCa_{3}\right]e^{-R{\left(L-L_{a}\right)}^{2}} \end{array}$$ (11)

$$\begin{array}{c} \frac{d[TSCa_{3}^{∼}]}{dt}=f\left[TSCa_{3}\right]e^{-R{\left(L-L_{a}\right)}^{2}}-g\left[TSCa_{3}^{∼}\right]+Z_{p}\left[TSCa_{3}^{*}\right]-Y_{p}\left[TSCa_{3}^{∼}\right] \end{array}$$ (12)

$$\begin{array}{c} \frac{d[TSCa_{3}^{*}]}{dt}=Y_{p}\left[TSCa_{3}^{∼}\right]-Z_{p}\left[TSCa_{3}^{*}\right]+Z_{r}\left[TS^{*}\right]{\left[Ca^{2+}\right]}^{3}-Y_{r}\left[TSCa_{3}^{*}\right] \end{array}$$ (13)

$$\begin{array}{c} \frac{d[TS^{*}]}{dt}=Y_{r}\left[TSCa_{3}^{*}\right]-Z_{r}\left[TS^{*}\right]{\left[Ca^{2+}\right]}^{3}+Z_{q}\left[TS^{∼}\right]-Y_{q}\left[TS^{*}\right] \end{array}$$ (14)

$$\begin{array}{c} \begin{array}{cc} \frac{d[TS^{∼}]}{dt} & =Y_{q}\left[TS^{*}\right]-Z_{q}\left[TS^{∼}\right]-g_{d}\left[TS^{∼}\right] \end{array} \end{array}$$ (15)

Note that to realize the cooperativity characteristics, three troponins are handled as one troponin system in the model. On the other hand, crossbridge forming state TSCa_3^∼^_ and TS^∼^ decreases to crossbridge unbound states TSCa_3_ and TS by the rate constant *g* and *g*_*d*_ whose values increases with crossbridge extension *h*_*w*_ − *h*_*wr*_. *X* [*μ*m] is the non-elastic portion of the contractile element and *L* − *X*_*w*_ and *L* − *X*_*p*_ are extensions of the attached crossbridges in the weak and power states, abbreviated as *h*_*w*_ and *h*_*p*_ (Eqs (7) and (8)), respectively. The time derivative of *X*_*p*_ and *X*_*w*_ are given by the products of the crossbridge sliding rate (*B*) and the crossbridge extension (*h*_*p*_ − *h*_*pr*_, *h*_*w*_ − *h*_*wr*_), which are different from the initial length(*h*_*pr*_[*μ*m], *h*_*wr*_[*μ*m]) shown in Eqs (9) and (10). Note that [$TSCa_{3}^{∼}$], [*TS*^∼^], [$TSCa_{3}^{*}$], [TS*], *h*_*p*_, and *h*_*w*_ are calculated using the equations in the original paper of the NL08 model [35] with modifications of *g* and *g*_*d*_ to be explained later.

The contraction time course is controlled by *Ca*^2+^, and *Ca*^2+^ release is controlled by the *Ca*^2+^ release equation. The release and absorption of *Ca*^2+^ by the sarcoplasmic reticulum (*Q*_*rel*_ [*μ*M/ms] and *Q*_*pump*_ [*μ*M/ms]), and also the changes in the *Ca*^2+^ in the NL08 model are expressed by the following equations.

$$\begin{array}{ccc} Q_{rel} & = & Q_{m}{\left(\frac{t}{t_{1}}\right)}^{4}e^{4(1-\frac{t}{t_{1}})}+Q_{pump\_rest} \end{array}$$ (16)

$$\begin{array}{ccc} Q_{pump} & = & K_{p}\frac{1}{1+{\left(\frac{K_{m}}{\left[Ca^{2+}\right]}\right)}^{2}} \end{array}$$ (17)

$$\begin{array}{ccc} \frac{d[Ca^{2+}]}{dt} & = & Q_{rel}-Q_{pump}-I_{troponin} \end{array}$$ (18)

$$\begin{array}{ccc} I_{troponin} & = & \left(\frac{d[TSCa_{3}]}{dt}+\frac{d[TSCa_{3}^{∼}]}{dt}+\frac{d[TSCa_{3}^{*}]}{dt}\right) \end{array}$$ (19)

Note that, *t* [ms] is the time parameter, [*Ca*^2+^] [*μ*M] is the concentration of *Ca*^2+^, *Q*_*m*_ [*μ*M/ms] is the maximum level of *Ca*^2+^ release, *t*_1_ [ms] is the interval to maximum *Q*_*rel*_, *Q*_*pump*_*rest*_ [*μ*M/ms] determines [*Ca*^2+^] at rest, *K*_*p*_ [*μ*M/ms] is the maximum value of *Q*_*pump*_, and *K*_*m*_ [*μ*M] is the value of [*Ca*^2+^] for *Q*_*pump*_ = *K*_*p*_/2. *I*_*troponin*_ represents the amount of *Ca*^2+^ bounds to the troponin system. Parameter values used in Eqs (16) and (17) are shown in Table 4.

**Table 4:** Parameters used in Eqs (16) and (17).

*Q*_*m*_ [*μ*M/ms]	*t*_1_ [ms]	*Q*_*pump*_*rest*_ [*μ*M/ms]	*K*_*p*_ [*μ*M/ms]	*K*_*m*_ [*μ*M]
3.2	8	0.03	0.15	0.2

The NL08 model is known to have a problem in the ejection phase, where *F*_*b*_ rapidly decreases when *L* shortens. As shown in Fig 4, *g* and *g*_*d*_ are the rate constants which determines the decreasing rate of crossbridges. Since these values increase with (*h*_*w*_ − *h*_*wr*_)^2^, TSCa_3_ and TSCa_3*_ decrease when $\left|\frac{dL}{dt}\right|$ increase which happens at both ejection and filling phase. To improve the filling phase characteristics, *K*_*γ*_ was introduced in [24], and we used this modification in our model as follows.

$$\begin{array}{ccc} g & = & Z_{a}+Y_{v}(1-e^{-\gamma_{m}{\left(h_{w}-h_{wr}\right)}^{2}}) \end{array}$$ (20)

$$\begin{array}{ccc} g_{d} & = & Y_{d}+Y_{c}{\left(L-L_{c}\right)}^{2}+Y_{vd}(1-e^{-\gamma_{m}{\left(h_{w}-h_{wr}\right)}^{2}}) \end{array}$$ (21)

$$\begin{array}{c} \;\gamma_{m}=\{\begin{array}{cc} \gamma \frac{1}{K_{\gamma }} & \frac{dX_{w}}{dt}>0\\ \gamma & otherwise \end{array} \end{array}$$ (22)

Note that, *h*_*wr*_ [*μ*m] is the steady state extension of the attached crossbridges in the weak state. *Z*_*a*_ [1/ms] and *Y*_*d*_ [1/ms] are crossbridge dissociation constants, *Y*_*v*_ [1/ms] and *Y*_*vd*_ [1/ms] are model parameters for the weakly-attached crossbridge extension, and *Y*_*c*_ [1/ms/*μ*m^2^], and *L*_*c*_ [*μ*m] are model parameters related to the half-sarcomere length.

As the characteristics of the end-diastolic pressure-volume relation (EDPVR) are similar in rats [36] and humans [37], by linearly scaling the force axis with the identical half-sarcomere length axis, we used the following mammalian exponential function as a human passive elastic force (*F*_*p*_ [mN/mm^2^]) model showing good agreement with the experimental data [38, 39]. The form of this equation was based on the equation used by Shim et al. [40] and Landesberg et al. [41].

$$\begin{array}{c} F_{p}=\{\begin{array}{cc} -K_{PL}(1-\frac{L}{L_{0}}) & L<L_{0}\\ K_{PE}(e^{D(\frac{L}{L_{0}}-1)}-1) & otherwise \end{array} \end{array}$$ (23)

Note that *L*_0_ [*μ*m] is resting half sarcomere length. *D*, *K*_*PL*_ [mN/mm^2^], and *K*_*PE*_ [mN/mm^2^] are constant parameters that determine the properties of the passive elastic component. The parameter values were manually adjusted to reproduce physiological human hemodynamics (Tables 5 and 6).

**Table 5:** Parameters in Eq (23).

*K*_*PE*_ [mN/mm^2^]	*K*_*PL*_ [mN/mm^2^]	*D*	*L*_0_ [*μ*m]
0.2692	7.561	28.08	1.043

Since *F*_*p*_ is usually measured using a piece of tissue or an LV cavity, we can consider that the characteristics of *F*_*p*_ are compatible with the macroscopic properties. On the other hand, since *F*_*b*_ is usually measured with a single cell or a small piece of ventricular fiber in which the effective cross-sectional area is difficult to measure, the measured force may contain large scale errors. We thus introduced a scale factor, *K*_*S*_, which is multiplied only with *F*_*b*_ to adjust the cellular contraction force. *K*_*S*_ was determined using the method proposed by Utaki et al. [24], which resulted in *K*_*S*_ = 7.24. Finally, LV wall tension *F*_*ext*_ [mN/mm^2^] in Eq (5) was calculated as follows.

$$\begin{array}{c} F_{ext}=K_{S}F_{b}+F_{p} \end{array}$$ (24)

**Table 6:** Parameters used to calculate cellular contraction force (*F*_*b*_).

*A*_*p*_	2700	[mN mm^−2^*μ*m^−1^*μ*M^−1^]
*A*_*w*_	540	[mN mm^−2^*μ*m^−1^*μ*M^−1^]
*h*_*pr*_	0.006	[*μ*m]
*h*_*wr*_	0.0001	[*μ*m]
*B*	0.5	[ms^−1^]
*Y*_*b*_	0.1816	[*μ*M^−1^*ms*^−1^]
*Y*_*c*_	1.0	[ms^−1^*μ*m^−2^]
*Y*_*d*_	0.0333	[ms^−1^]
*Y*_*p*_	0.1397	[ms^−1^]
*Y*_*q*_	0.2328	[ms^−1^]
*Y*_*r*_	0.1397	[ms^−1^]
*Y*_*v*_	1.5	[ms^−1^]
*Y*_*vd*_	1.5	[ms^−1^]
*Z*_*a*_	0.0023	[ms^−1^]
*Z*_*b*_	0.1397	[ms^−1^]
*Z*_*p*_	0.2095	[ms^−1^]
*Z*_*q*_	0.3724	[ms^−1^]
*Z*_*r*_	7.2626	[*μM*^3^ms^−1^]
*L*_*c*_	1.2	[*μ*m]
*K*_*γ*_	85.3	
*γ*	28000	[*μm*^−2^]
*f*	0.0023	[*ms*^−1^]
*R*	15	[*μm*^−2^]
*L*_*a*_	1.15	[*μm*]

### Mathematical definitions of elastance

- instantaneous elastance
  Considering LV as one elastic compartment, the instantaneous elastance *E*_*inst*_ can be defined by the ratio between the instantaneous changes in pressure (*P*_*lv*_) and small changes in volume (*V*_*lv*_), as can be defined as follows.
  $$\begin{array}{c} E_{inst}=\frac{dP_{lv}}{dV_{lv}} \end{array}$$ (25)
- load-dependent elastance
  For the heart, pressure and the volume follow a different pressure-volume curve if the afterload is different. Usually, the end-systolic pressure volume curve is measured by measuring several pressure-volume curves with different loads; thus, we can define load-dependent elastance *E*_*load*_ by the ratio between the changes in pressure with changes in afterload $\left(\frac{dP_{lv}}{dR_{out}}\right)$ and volume changes with the changes in the afterload $\left(\frac{dV_{lv}}{dR_{out}}\right)$ as follows.
  $$\begin{array}{c} E_{load}=\frac{\frac{dP_{lv}}{dR_{out}}}{\frac{dV_{lv}}{dR_{out}}} \end{array}$$ (26)

## Simulation

### Simulation conditions

Simulation program was written in C, and performed in 0.01 [ms] intervals until a periodic limit cycle was achieved, requiring around 100 cardiac cycles. In this study, during the simulation of 100 cardiac cycle to achive periodic limit cycle and following measurement of elastance, when *P*_*lv*_ exceeded 70.0 [mmHg] during the isovolumic contraction phase, *P*_*a*_ and *V*_*a*_ were fixed to constant values of 70.0 [mmHg] and 105.0 [mL], respectively, which corresponds to the onset condition of ejection phase. In addition, *P*_*lv*_ after the onset time of ejection rapidly changed to 70.0 [mmHg], and *V*_*lv*_ also rapidly changed to 126.419 [mL]. We also measured the *E*_*inst*_ by decreasing the *V*_*lv*_ by 0.25% (*ΔV*_*lv*_) at a certain time in the ejection phase and by evaluating the pressure drop (*ΔP*_*lv*_) in the next time interval. Thus, *E*_*inst*_ was calculated by *ΔP*_*lv*_/*ΔV*_*lv*_.

### Simulation results

**Fig 6:**
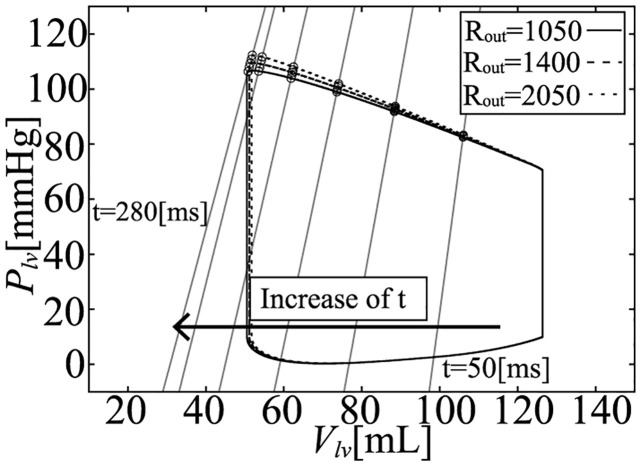
PVloops and isochronous *P*_*lv*_—*V*_*lv*_ relations.

**Fig 7:**
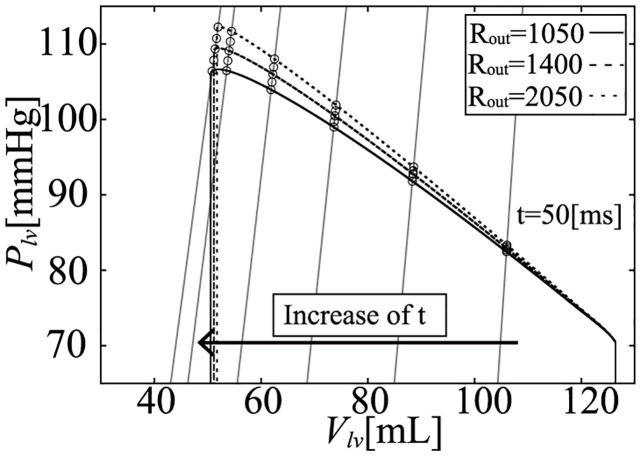
Isochronous *P*_*lv*_—*V*_*lv*_ relations during the ejection phase.

**Fig 8:**
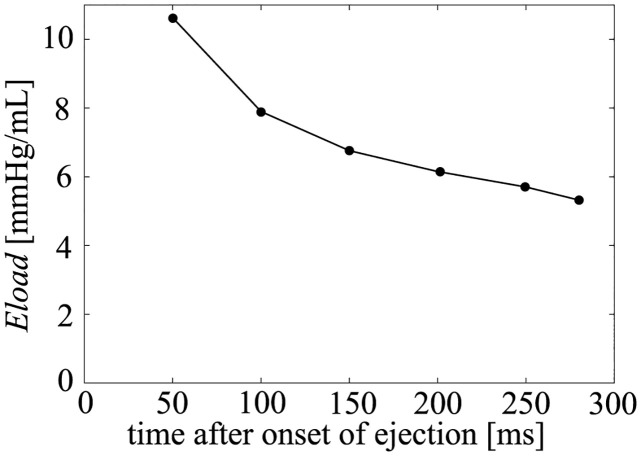
Changes in *E*_*load*_ with time.

**Fig 9:**
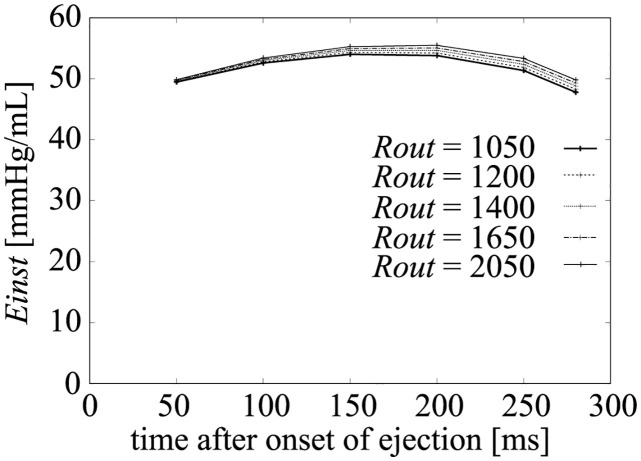
Changes in *E*_*inst*_ with time.

To evaluate the influence of the afterload on pressure (*P*_*lv*_) and volume (*V*_*lv*_), we performed a simulation with several peripheral resistance values: *R*_*out*_ = 1050, 1200, 1400, 1650, and 2050 [mmHg ⋅ ms/mL]. Under these conditions, the PV loops are shown in Fig 6 and the enlarged ejection phase in Fig 7. In both figures, the isochronous points of *P*_*lv*_—*V*_*lv*_ are shown at 50, 100, 150, 200, 250, and 280 [ms] after the onset time of the ejection, and the isochronous linear approximation lines of each time point are also shown in these figures. The values of each slope (*E*_*load*_) are shown in Fig 8, and *E*_*load*_, *V*_*lv*_-axis intercepts (*V*_0_), and *R*^2^ of these lines are shown in Table 7. From the results, the isochronous lines had high *R*^2^ values, thus the isochronous points of *P*_*lv*_—*V*_*lv*_ had high linearity during the ejection phase. We also noted that *V*_0_ greatly decreased with time, but after the initial drop, *E*_*load*_ slightly decreased over time, although the amount of decrease was quite small as we can see in Fig 7. With the instantaneous volume drop simulation, we also measured *E*_*inst*_ (Fig 9) at the same time points shown in Fig 8 with *R*_*out*_ = 1050, 1200, 1400, 1650, 2050. As you can find in Fig 9, *E*_*inst*_ slightly increased and decreased during the ejection phase.

**Table 7:** Changes in *E*_*inst*_, *E*_*load*_, *V*_0_ with time, and its *R*^2^.

*t* [ms]	*E*_*inst*_ (*R*_*out*_ = 1050)	*E*_*load*_	*V*_0_	*R* ^2^
50	49.53	10.6280	98.2267	0.999997
100	52.62	7.8969	76.7246	0.999989
150	54.04	6.7593	58.9819	0.999977
200	53.83	6.1503	44.9864	0.999963
250	51.37	5.7014	34.8865	0.999956
280	47.78	5.3194	30.8502	0.999953

**Fig 10:**
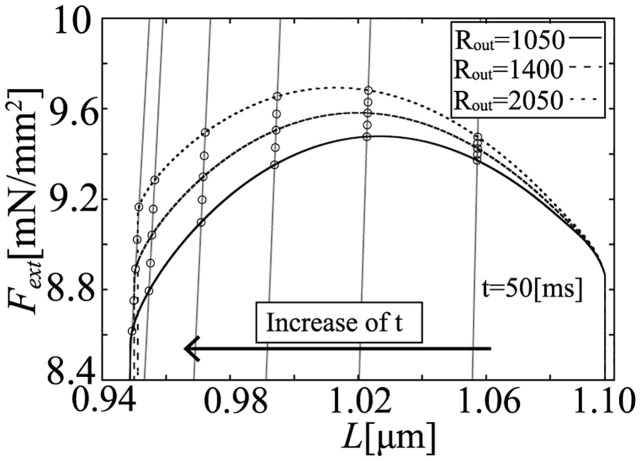
Isochronous *F*_*ext*_—*L* relation during the ejection phase.

In the model, the relation between volume (*V*_*lv*_) and half-sarcomere length (*L*) is linear, as shown in Eqs (2) and (3). On the other hand, although the relation between LV pressure (*P*_*lv*_) and wall tension (*F*_*ext*_) is nonlinear, as shown in Eq (5), it can be approximated as linear for a small change of *P*_*lv*_. Thus, if we convert the isochronous relation of *P*_*lv*_—*V*_*lv*_ in Fig 7 into the isochronous relation of *F*_*ext*_—*L* shown in Fig 10, the relation remains linear. The linear approximations for the isochronous points of *F*_*ext*_—*L* have high-linearity, as evidenced by *R*^2^ in Table 8. The slopes (*k*_*a*_) of these lines slightly decreased, but they appear to be almost parallel.

**Table 8:** Changes in the slope of the isochronous *F*_*ext*_—*L* relation (*k*_*a*_) with time and its *R*^2^.

*t* [ms]	*k*_*a*_	*R* ^2^
50	642.82	0.999997
100	445.07	0.999988
150	357.38	0.999974
200	307.93	0.999956
250	274.58	0.999939
280	254.25	0.999936

## Results

### Elastance of simplified hemodynamic model

From the simulation results, it can be seen that *E*_*inst*_ slightly increased and then decreased, and its time course was similar to that of [*Ca*^2+^]. On the other hand, *E*_*load*_ was markedly different from *E*_*inst*_ at each time point. *E*_*load*_ initially decreased by around 20%, and decreased slightly over time. On the other hand, if we measure *E*_*inst*_ under different *R*_*out*_ (Fig 9), we can find that the values at the same time points are very close. For the measurement of *E*_*load*_, we have to change *R*_*out*_, however, from Fig 7, we can find that the isochronous PV points lie on a line, that means that the slope is constant and does not depend on *R*_*out*_, which is similar with the characteristics of *E*_*max*_ which is considered to be a good index of contractility because of its independence with respect to afterload. Thus we can say that at a certain time point, both elastances were independent of the afterload (*R*_*out*_). The mathematical reasons for these findings are discussed in the following section.

### *E*_*inst*_ in the simplified hemodynamic model

*E*_*inst*_ was mathematically defined by Eq (25), and by using this model, we can represent *E*_*inst*_ as follows.

$$\begin{array}{c} \begin{array}{c} E_{inst}=\frac{dP_{lv}}{dV_{lv}}=\frac{\partial P_{lv}}{\partial R_{lv}}\frac{dR_{lv}}{dV_{lv}}+\frac{\partial P_{lv}}{\partial F_{ext}}\frac{dF_{ext}}{dV_{lv}} \end{array} \end{array}$$ (27)

From Eq (5), the partial derivative $\frac{\partial P_{lv}}{\partial R_{lv}}$ can be derived as follows.

$$\begin{array}{c} \begin{array}{c} \frac{\partial P_{lv}}{\partial R_{lv}}=-\frac{2K_{u}F_{ext}h_{lv}}{R_{lv}^{2}} \end{array} \end{array}$$ (28)

Derivative $\frac{dR_{lv}}{dV_{lv}}$ can be derived from Eq (2) as follows.

$$\begin{array}{c} \frac{dR_{lv}}{dV_{lv}}=K_{R} \end{array}$$ (29)

Similarly, from Eq (5), the partial derivative $\frac{\partial P_{lv}}{\partial F_{ext}}$ can be derived as follows.

$$\begin{array}{c} \begin{array}{c} \frac{\partial P_{lv}}{\partial F_{ext}}=\frac{2K_{u}h_{lv}}{R_{lv}} \end{array} \end{array}$$ (30)

From Eqs (2) and (3) and (24), the derivative $\frac{dF_{ext}}{dV_{lv}}$ can be derived as follows.

$$\begin{array}{c} \begin{array}{c} \frac{dF_{ext}}{dV_{lv}}=K_{L}K_{R}(K_{S}\frac{dF_{b}}{dL}+\frac{dF_{p}}{dL}) \end{array} \end{array}$$ (31)

The elastance of the cellular contraction force term $\frac{dF_{b}}{dL}$ can be derived by differentiating Eq (6) with *L* as follows.

$$\begin{array}{c} \frac{dF_{b}}{dL}=A_{w}(\left[TSCa_{3}^{∼}\right]+\left[TS^{∼}\right])+A_{p}(\left[TSCa_{3}^{*}\right]+\left[TS^{*}\right]) \end{array}$$ (32)

Also, by differentiating Eq (23) with *L*, $\frac{dF_{p}}{dL}$ can be derived as follows.

$$\begin{array}{c} \frac{dF_{p}}{dL}=\{\begin{array}{cc} \frac{K_{PL}}{L_{0}} & L<L_{0}\\ \frac{K_{PE}D}{L_{0}}(e^{D(\frac{L}{L_{0}}-1)}) & otherwise \end{array} \end{array}$$ (33)

Although Eqs (32) and (33) appear in Eq (31), from the simulation results, $K_{S}\frac{dF_{b}}{dL}$ was about 100 times larger than $\frac{dF_{p}}{dL}$. Thus, we can neglect the $\frac{dF_{p}}{dL}$ term in Eq (31).

In this model, *E*_*inst*_ is denoted by Eq (27). From the simulation results, we found that the second term of the right-hand side of Eq (27) was dominant. The second term of the right-hand side of Eq (27) is the product of Eqs (30) and (31), that represents the changes in the LV pressure by the changes in the LV wall tension, and the changes in the LV wall tension by the changes in the LV volume, respectively. Since $K_{S}\frac{dF_{b}}{dL}$ term was dominant in Eq (31) and $\frac{dF_{b}}{dL}$ is considered to be proportional to the attached cross bridges from Eq (32), finally, *E*_*inst*_ can be approximated by the following equation (Eq (34)).

$$\begin{array}{c} E_{inst}\approx \frac{2K_{u}h_{lv}K_{L}K_{R}K_{S}A_{p}(\left[TSCa_{3}^{*}\right]+\left[TS^{*}\right])}{R_{lv}} \end{array}$$ (34)

**Fig 11:**
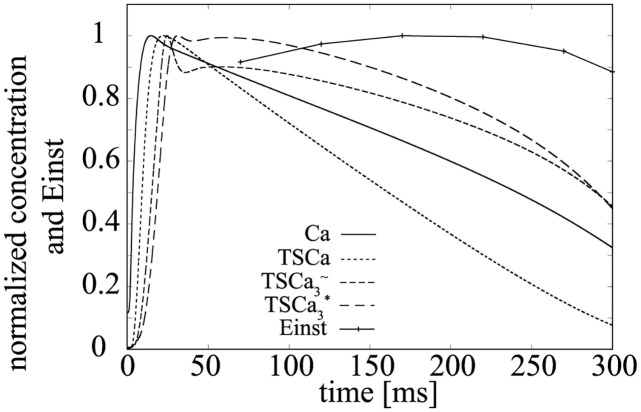
Normalized concentration of [*Ca*^2+^], [*TSCa*_3_], $\left[TSCa_{3}^{∼}\right]$, $\left[TSCa_{3}^{*}\right]$ and *E*_*inst*_.

In this model, $\left[TSCa_{3}^{*}\right]$ increases with $\left[TSCa_{3}^{∼}\right]$, and $\left[TSCa_{3}^{∼}\right]$ increases with [*TSCa*_3_], and [*TSCa*_3_] increases with [*TS*] × [*Ca*^2+^]. Thus, as we can find from the changes in these normalized concentrations shown in Fig 11, $\left[TSCa_{3}^{*}\right]$ tends to follow changes in [*Ca*^2+^]. And since [TS*] is small, *E*_*inst*_ can be understood as the LV wall tension elastance mainly determined by the *Ca*^2+^ concentration.

### *E*_*load*_ in the simplified hemodynamic model

*E*_*load*_ was mathematically defined as Eq (26). Although the simulation conditions were not physiological, we fixed *P*_*lv*_ at the onset time of ejection to simplify the mathematical analysis. The phenomena in the ejection phase can be divided into two steps: 1) initial phase and 2) steady state transition during the ejection phase.

step 1 Immediately after the onset time of ejection, transient variations in crossbridge extension changes occur in short time periods, depending on the afterload.
step 2 The linear isochrones of the *P*_*lv*_—*V*_*lv*_ relations remain linear during the ejection phase with small changes in their slopes.

#### step 1

It is known that the response to transient force is observed in the initial phase of isovelocity-shortening experiments using skeletal muscles. However, the phenomenon is difficult to measure under accurate isovelocity conditions in real experiments [42–45]. At this phase, the contraction force changes significantly depending on the velocity. In our analysis, as *P*_*lv*_ at the onset time of ejection was fixed, the shortening velocity of the half-sarcomere length ($\frac{dL}{dt}$) becomes roughly proportional to the inverse of the afterload (*R*_*out*_), corresponding to the quasi isovelocity-shortening condition. Thus, we can predict that the variation of the initial cellular contraction force resulting from the variations of the afterload is caused by the same mechanism.

**Fig 12:**
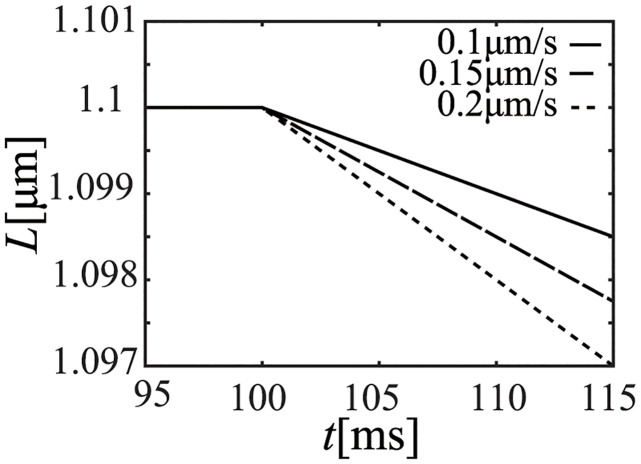
Time course of half-sarcomere length (*L*).

**Fig 13:**
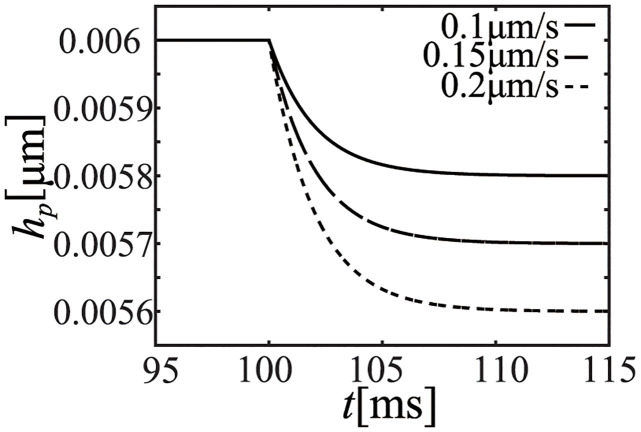
Time course of the crossbridge length (*h*_*p*_).

**Fig 14:**
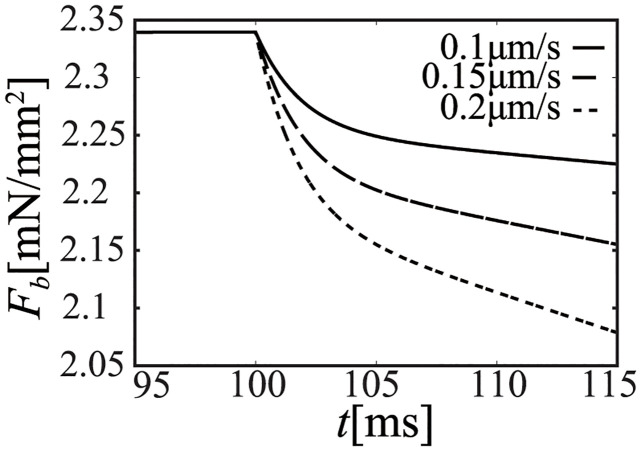
Time course of the cellular contraction force (*F*_*b*_).

We performed an isovelocity-shortening simulation by using the NL08 model under the condition of the onset time of ejection. Since the effect of variations in *R*_*out*_ to [*Ca*^2+^] is small, [*Ca*^2+^] at the time of ejection can be considered as constant for this short period, even if the afterload (*R*_*out*_) is different. Thus, we fixed [*Ca*^2+^] as 0.3 [*μ*M], and performed an isovelocity-shortening simulation of the half-sarcomere length ($\frac{dL}{dt}$) with shortening velocities of 0.1, 0.15, and 0.20 [*μ*m/s] corresponding to the condition close to the hemodynamics simulation afterloads. The time courses of *L*, *h*_*p*_, and *F*_*b*_ are shown in Figs 12, 13 and 14, respectively. Note that the isovelocity condition starts at *t* = 100 [ms] in these figures.

As shown in Figs 13 and 14, crossbridge length (*h*_*p*_) of the power state becomes constant after an initial decrease and the time course of *F*_*b*_ is similar to that of *h*_*p*_.

From the model equations, the shortening velocity of the half-sarcomere length ($\frac{dL}{dt}$) was found to be inversely proportional to the afterload (*R*_*out*_). Thus, *h*_*p*_ changes depending on Eqs (8) and (9)as follows.

$$\begin{array}{c} \frac{dh_{p}}{dt}=\frac{dL}{dt}-\frac{dX_{p}}{dt}=\frac{dL}{dt}-B\left(h_{p}-h_{pr}\right) \end{array}$$ (35)

After the initial decrease of *h*_*p*_, it exponentially got close to the steady state length *h*_*p*,*ss*_, which can be calculated from $\frac{dh_{p}}{dt}=0$ as follows.

$$\begin{array}{c} h_{p,ss}=\frac{1}{B}\frac{dL}{dt}+h_{pr} \end{array}$$ (36)

From this equation, *h*_*p*_ decreased from the resting crossbridge length (*h*_*pr*_) to the steady state length that is linearly related with the shortening velocity of the half-sarcomere length ($\frac{dL}{dt}$). This is what produces the well-known characteristics of the force-velocity relation. Thus, if the troponin system probabilities are constant, the cellular contraction force (*F*_*b*_) is proportional to *h*_*p*_, which is also proportional to $\frac{dL}{dt}$. As a result, the isochronous *F*_*ext*_—*L* (≈*F*_*b*_—*L*) points located at the same position of the onset time of ejection, move in the *L* direction, depending on the velocity ($\frac{dL}{dt}$) determined by the afterload. However, the movements done in the *F*_*b*_ direction are maintained by the relation between $\frac{dF_{b}}{dt}$ and $\frac{dL}{dt}$, which can be approximated with a linear relation as shown in Eq (35). Thus, the initial cellular contraction force and length relation becomes almost linear.

If we approximate the initial transient changes in $\frac{dL}{dt}$ and $\frac{dF_{ext}}{dt}$ for duration *Δt* as linear with respect to time, by denoting $\frac{dL}{dt}$ at time *Δt* after onset of ejection as ${\dot{L}}_{\Delta t}$, the changes of *L* and *F*_*ext*_, *ΔL* and *ΔF*_*ext*_ can be approximated as follows.

$$\begin{array}{c} \Delta L\approx \frac{1}{2}{\dot{L}}_{\Delta t}\Delta t \end{array}$$ (37)

$$\begin{array}{c} \Delta F_{ext}\approx A_{p}\left(\left[TSCa_{3}^{*}\right]+\left[TS^{*}\right]\right)(\frac{1}{B}{\dot{L}}_{\Delta t}) \end{array}$$ (38)

Then, the initial slope between *P*_*lv*_ and *V*_*lv*_ which can be considered as initial *E*_*load*_ is approximated by the following eqution.

$$\begin{array}{c} E_{load,init}=\frac{\Delta P_{lv}}{\Delta V_{lv}}=\frac{2K_{u}h_{lv}\Delta F_{b}}{R_{lv}}\frac{K_{R}K_{L}}{\Delta L} \end{array}$$ (39)

$$\begin{array}{c} \approx \frac{K_{L}K_{R}K_{u}h_{lv}A_{p}(\left[TSCa_{3}^{*}\right]+\left[TS^{*}\right])}{R_{lv}B\Delta t} \end{array}$$ (40)

In the simulation results in section Simulation results, the shortening velocity of the half-sarcomere length ($\frac{dL}{dt}$) was affected by the cellular contraction force (*F*_*b*_); thus, its ratio was slightly different from the inverse of the afterload (*R*_*out*_). However, the difference between *h*_*p*_ and *h*_*p*,*ss*_ was around 2.3% at 15 [ms] after the onset time of ejection.

#### step 2

As explained in the previous section, the fixed *F*_*ext*_—*L* points with different afterloads (i.e. fixed *P*_*lv*_—*V*_*lv*_ points) proceeded to isochrones immediately after the onset time of ejection.

We assume that the relation between *F*_*ext*_ and *L* is linear, that is *F*_*ext*_ = *k*_*a*_*L*+ *k*_*b*_ at a certain time *t*_0_ during the ejection phase. If we assume that linearity is maintained after the small period (*Δt*), then the following equation holds.

$$\begin{array}{c} \begin{array}{cc} F_{ext} & +\int_{t_{0}}^{t_{0}+\Delta t}\frac{dF_{ext}}{dt}dt\\ & =(k_{a}+\int_{t_{0}}^{t_{0}+\Delta t}\frac{dk_{a}}{dt}dt)(L+\int_{t_{0}}^{t_{0}+\Delta t}\frac{dL}{dt}dt)+k_{b}+\int_{t_{0}}^{t_{0}+\Delta t}\frac{dk_{b}}{dt}dt \end{array} \end{array}$$ (41)

In the time derivative form, the following equation holds.

$$\begin{array}{c} \frac{dF_{ext}}{dt}=(k_{a}+2\int_{t_{0}}^{t_{0}+\Delta t}\frac{dk_{a}}{dt}dt)\frac{dL}{dt}+\frac{dk_{a}}{dt}(L+2\int_{t_{0}}^{t_{0}+\Delta t}\frac{dL}{dt}dt)+\frac{dk_{b}}{dt} \end{array}$$ (42)

Here, we introduce the assumption that changes in the slope of *k*_*a*_ from the *F*_*ext*_—*L* relation are small and can be neglected in a short time period, i.e., $\frac{dk_{a}}{dt}\approx 0$; then, the following equation holds.

$$\begin{array}{c} \begin{array}{c} \frac{dF_{ext}}{dt}\approx k_{a}\frac{dL}{dt}+\frac{dk_{b}}{dt} \end{array} \end{array}$$ (43)

If we assume that the increase in *F*_*ext*_ has term proportional to *L* as follow, the resulting *F*_*ext*_—*L* relation still becomes linear with time.

$$\begin{array}{c} \begin{array}{c} \frac{dF_{ext}}{dt}\approx k_{a}\frac{dL}{dt}+\frac{dk_{b}}{dt}+k_{c}L \end{array} \end{array}$$ (44)

Thus, if the model equations can be decomposed into the above equation form, we can say that the model itself has in its nature the property of a linear *F*_*ext*_—*L* relation.

First, by temporally differentiating the LV wall tension (Eq (24)), we get $\frac{dF_{ext}}{dt}$ as follows.

$$\begin{array}{c} \begin{array}{c} \frac{dF_{ext}}{dt}=K_{S}\frac{dF_{b}}{dt}+\frac{dF_{p}}{dt} \end{array} \end{array}$$ (45)

$$\begin{array}{c} \frac{dF_{b}}{dt}=T_{1}+T_{2}+T_{3}+T_{4} \end{array}$$ (46)

$$\begin{array}{c} \begin{array}{c} T_{1}=A_{w}(\frac{d[TSCa_{3}^{∼}]}{dt}+\frac{d[TS^{∼}]}{dt})h_{w} \end{array} \end{array}$$ (47)

$$\begin{array}{c} \begin{array}{c} T_{2}=A_{w}(\left[TSCa_{3}^{∼}\right]+\left[TS^{∼}\right])(\frac{dL}{dt}-\frac{dX_{w}}{dt}) \end{array} \end{array}$$ (48)

$$\begin{array}{c} \begin{array}{c} T_{3}=A_{p}(\frac{d[TSCa_{3}^{*}]}{dt}+\frac{d[TS^{*}]}{dt})h_{p} \end{array} \end{array}$$ (49)

$$\begin{array}{c} \begin{array}{c} T_{4}=A_{p}(\left[TSCa_{3}^{*}\right]+\left[TS^{*}\right])(\frac{dL}{dt}-\frac{dX_{p}}{dt}) \end{array} \end{array}$$ (50)

$$\begin{array}{c} \frac{dF_{p}}{dt}=\{\begin{array}{cc} \frac{K_{PL}}{L_{0}}\frac{dL}{dt} & L<L_{0}\\ \frac{K_{PE}D}{L_{0}}(e^{D(\frac{L}{L_{0}}-1)})\frac{dL}{dt} & otherwise \end{array} \end{array}$$ (51)

In Eq (51), if *L* < *L*_0_, then $\frac{dF_{p}}{dt}$ is proportional to $\frac{dL}{dt}$. If *L* ≥ *L*_0_, then $\frac{dF_{p}}{dt}$ is a function of *L* and $\frac{dL}{dt}$. However, from the simulation results, changes in *L* with *R*_*out*_ is 0.63%. Thus, we approximated $\frac{dF_{p}}{dt}\propto \frac{dL}{dt}$, and the passive elastic force component is approximated by using Eq (44).

On the other hand, in Eq (46), $\frac{dF_{b}}{dt}$ is composed of four terms. If we look at these terms, *T*_1_ and *T*_2_ are similar to *T*_3_ and *T*_4_, respectively; thus, the mathematical analysis of *T*_1_ and *T*_2_ becomes the same for *T*_3_ and *T*_4_, respectively. Moreover, *A*_*p*_ is five times larger than *A*_*w*_. Therefore, we can reduce our analysis of Eq (46) to the analysis of *T*_3_ and *T*_4_.

*T*_3_ is a product of the crossbridge length (*h*_*p*_) and the time derivative term of $\left[TSCa_{3}^{*}\right]+\left[TS^{*}\right]$. As explained in section step 1, *h*_*p*_ decreases from the resting crossbridge length (*h*_*pr*_), and the amount of decrease is proportional to the shortening velocity of the half-sarcomere length $\left(\frac{dL}{dt}\right)$. However, as we can see from Fig 13, the decrease of *h*_*p*_ is so small that, *h*_*p*_ can be approximated by *h*_*pr*_. In addition, the time derivative of $\left[TSCa_{3}^{*}\right]+\left[TS^{*}\right]$ is affected by *L* and $\frac{dL}{dt}$. The effect of *L* comes from Eq (11) where the term can be approximated to be linear with *L* in the short time period. In general, *h*_*p*_ can be approximated to *h*_*pr*_ and then *F*_*b*_ becomes proportional to $\left[TSCa_{3}^{*}\right]+\left[TS^{*}\right]$. As the time derivative of $\left[TSCa_{3}^{*}\right]+\left[TS^{*}\right]$ is approximated to be proportional to the value itself, *T*_3_ can be approximated to be linear with *F*_*b*_.

*T*_4_ is a product of $\left[TSCa_{3}^{*}\right]+\left[TS^{*}\right]$ and the time derivative of *h*_*p*_. As mentioned above, the power state ($\left[TSCa_{3}^{*}\right]+\left[TS^{*}\right]$) can be approximated to be linear with *F*_*b*_. The time derivative of *h*_*p*_ can be deformed as follows.

$$\begin{array}{c} \frac{dh_{p}}{dt}=\frac{dL}{dt}-\frac{dX_{p}}{dt}=\frac{1}{B}\frac{d^{2}X_{p}}{dt^{2}} \end{array}$$ (52)

As explained in section step 1, except for the initial ∼15 [ms] after the onset time of ejection, Eq (52) reaches its steady state. This means that $\frac{d^{2}X_{p}}{dt^{2}}$ can be approximated as a small constant. As the value of *F*_*p*_ is under 2% and increases to 8% of *F*_*ext*_ during the ejection phase, we approximate *F*_*b*_ as *F*_*ext*_. Afterward, by using *K*_1_, *K*_2_ and *K*_3_ as constants, the following equation holds.

$$\begin{array}{c} \frac{dF_{ext}}{dt}\approx K_{1}F_{b}+K_{2}+K_{3}L\approx K_{1}F_{ext}+K_{2}+K_{3}L \end{array}$$ (53)

Afterward, by using Eqs (3) and (2), the time derivative of the half-sarcomere length ($\frac{dL}{dt}$) can be derived as follows.

$$\begin{array}{c} \frac{dL}{dt}=K_{L}K_{R}\frac{dV_{lv}}{dt} \end{array}$$ (54)

Here, the sum of the LV volume (*V*_*lv*_) and the aortic volume (*V*_*a*_) during the ejection phase is denoted as the total volume (*V*_*tot*_).

$$\begin{array}{c} V_{tot}=V_{lv}+V_{a} \end{array}$$ (55)

Subsequently, $\frac{dL}{dt}$ can be represented as follows.

$$\begin{array}{c} \begin{array}{c} \frac{dL}{dt}=K_{L}K_{R}(\frac{\partial V_{lv}}{\partial V_{tot}}\frac{dV_{tot}}{dt}+\frac{\partial V_{lv}}{\partial F_{ext}}\frac{dF_{ext}}{dt}) \end{array} \end{array}$$ (56)

During the ejection phase, the time derivative of the total volume ($\frac{dV_{tot}}{dt}$) corresponds to the blood flow from the aorta to the periphery, which can be represented as follows.

$$\begin{array}{c} \frac{dV_{tot}}{dt}=-\frac{P_{a}-P_{E2}}{R_{out}} \end{array}$$ (57)

Moreover, as aortic resistance (*R*_*lo*_) is small, the following approximation holds.

$$\begin{array}{c} P_{lv}\approx P_{a} \end{array}$$ (58)

Now, $\frac{dV_{tot}}{dt}$, which is the first term on the right-hand side of Eq (56), can be approximated as follows.

$$\begin{array}{c} \frac{dV_{tot}}{dt}\approx -\frac{P_{lv}-P_{E2}}{R_{out}}=-\frac{\frac{2K_{u}F_{ext}h_{lv}}{K_{R}V_{lv}+K_{V}}-P_{E2}}{R_{out}} \end{array}$$ (59)

In addition, from Eqs (1), (55) and (58), the total volume (*V*_*tot*_) can be approximated as follows.

$$\begin{array}{c} V_{tot}\approx V_{lv}+C_{a}P_{lv} \end{array}$$ (60)

From Eq (60), $\frac{\partial V_{lv}}{\partial V_{tot}}$ in the first term on the right-hand side of Eq (56) can be obtained as follows.

$$\begin{array}{c} \begin{array}{c} \frac{\partial V_{lv}}{\partial V_{tot}}\approx \frac{1}{1+C_{a}\frac{\partial P_{lv}}{\partial V_{lv}}} \end{array} \end{array}$$ (61)

Similarly, by differentiating Eq (60) with the LV wall tension (*F*_*ext*_), $\frac{\partial V_{lv}}{\partial F_{ext}}$ that appears in the second term on the right-hand side of Eq (56) can be obtained as follows.

$$\begin{array}{c} \begin{array}{c} \frac{\partial V_{lv}}{\partial F_{ext}}\approx \frac{-C_{a}\frac{\partial P_{lv}}{\partial F_{ext}}}{1+C_{a}\frac{\partial P_{lv}}{\partial V_{lv}}} \end{array} \end{array}$$ (62)

Here, the first term on the right-hand side of Eq (56) is the product of Eqs (61) and (59). Since $C_{a}\frac{\partial P_{lv}}{\partial V_{lv}}$ in Eq (61) becomes around -0.07, the changes in $\frac{\partial V_{lv}}{\partial V_{tot}}$ with respect to changes in *F*_*ext*_ can be neglected. While Eq (59) consists of the component proportional to *F*_*ext*_ and constant. Thus the first term on the right-hand side of Eq (56) can be approximated as follows.

$$\begin{array}{ccc} & & \frac{\partial V_{lv}}{\partial V_{tot}}\frac{dV_{tot}}{dt}\approx -\frac{1}{1+C_{a}\frac{\partial P_{lv}}{\partial V_{lv}}}\frac{\frac{2K_{u}F_{ext}h_{lv}}{K_{R}V_{lv}+K_{V}}-P_{E2}}{R_{out}}\\ & & =-\frac{1}{1+\frac{2C_{a}K_{U}h_{lv}K_{R}F_{ext}}{{\left(K_{R}V_{lv}+K_{V}\right)}^{2}}}(\frac{2K_{u}h_{lv}F_{ext}}{(K_{R}V_{lv}+K_{V})R_{out}}-\frac{P_{E2}}{R_{out}})\\ & & \approx -\frac{1}{R_{out}}(\frac{2K_{u}h_{lv}}{K_{R}V_{lv}+K_{V}}F_{ext}-P_{E2}) \end{array}$$ (63)

On the other hand, the second term on the right-hand side of Eq (56) is the product of Eq (62) and $\frac{dF_{ext}}{dt}$. As explained above, since $C_{a}\frac{\partial P_{lv}}{\partial V_{lv}}$ in Eq (62) can be neglected, and $\frac{\partial P_{lv}}{\partial F_{ext}}=\frac{2K_{u}h_{lv}}{K_{R}V_{lv}+K_{V}}$ becomes constant with respect to *F*_*ext*_, this term can be approximated as proportional to $\frac{dF_{ext}}{dt}$, as changes in other terms are small with respect to changes in *F*_*ext*_ and *L*. Thus the second term of right-hand side of Eq (56) can be approximated as follows.

$$\begin{array}{ccc} & & \frac{\partial V_{lv}}{\partial F_{ext}}\frac{dF_{ext}}{dt}\approx -\frac{2C_{a}K_{u}h_{lv}}{K_{R}V_{lv}+K_{V}}\frac{dF_{ext}}{dt} \end{array}$$ (64)

In summary, the following approximation holds with the constants *K*_4_, *K*_5_, and *K*_6_.

$$\begin{array}{ccc} \frac{dL}{dt} & \approx & -\frac{2K_{L}K_{R}K_{u}h_{lv}}{\left(K_{R}V_{lv}+K_{V}\right)R_{out}}F_{ext}+\frac{K_{L}K_{R}P_{E2}}{R_{out}}-\frac{2C_{a}K_{L}K_{R}K_{u}h_{lv}}{K_{R}V_{lv}+K_{V}}\frac{dF_{ext}}{dt}\\ & & =K_{4}F_{ext}+K_{5}+K_{6}\frac{dF_{ext}}{dt} \end{array}$$ (65)

By using this equation and Eq (53), the following equation holds.

$$\begin{array}{ccc} \frac{dF_{ext}}{dt} & \approx & \frac{K_{1}}{K_{4}+K_{1}K_{6}}\frac{dL}{dt}-\frac{K_{2}K_{4}+K_{1}K_{5}}{K_{4}+K_{1}K_{6}}-\frac{K_{4}K_{3}}{K_{4}+K_{1}K_{6}}L \end{array}$$ (66)

Therefore, except for the initial transient period, the relation between $\frac{dL}{dt}$ and $\frac{dF_{ext}}{dt}$ approximately satisfies Eq (44) during the ejection phase. This occurs under the assumption that the relation between *L* and *F*_*ext*_ satisfies *F*_*ext*_ = *k*_*a*_*L* + *k*_*b*_.

**Fig 15:**
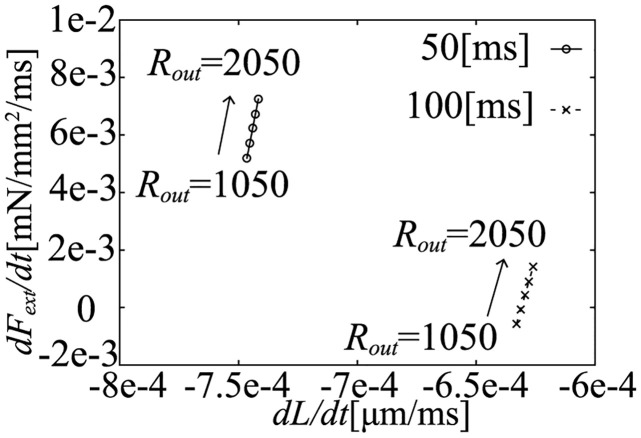
The relation between $\frac{dF_{ext}}{dt}$ and $\frac{dL}{dt}$ at 50, 100 [ms] after the onset time of ejection.

**Fig 16:**
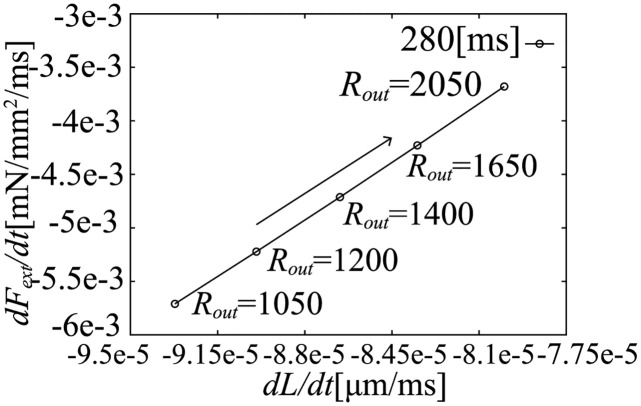
The relation between $\frac{dF_{ext}}{dt}$ and $\frac{dL}{dt}$ at 280 [ms] after the onset time of ejection.

In the simulation results at 50 and 100 [ms] after the onset time of ejection, we can find a highly linear relation between $\frac{dL}{dt}$ and $\frac{dF_{ext}}{dt}$, as shown in Fig 15. Similarly, at 280 [ms] after the onset time of ejection, we also can find a highly linear relation as shown in Fig 16.

Thus, from the above analysis, we can understand why the isochronous LV wall tension (*F*_*ext*_)—half-sarcomere length (*L*) relation remains linear under the variation of the afterload (*R*_*out*_) during the ejection phase; that is, the characteristics come from the combination of the characteristics of the cardiac muscle and compliance of the LV and aorta.

### Elastance of the time-varying elastance model (TVEM)

The Time Varying Elastance Model (TVEM) is one of the major simple LV model often used in hemodynamic models. In the model, a time-dependent elastic function (*E*(*t*)) and the constant zero-pressure fill volume *V*_0_ are used to relate LV pressure and LV volume as follows.

$$\begin{array}{c} P_{lv}=E\left(t\right)\left(V_{lv}-V_{0}\right) \end{array}$$ (67)

Here, we used the following function for *E*(*t*) proposed by Heldt et al. [26].

$$\begin{array}{c} E\left(t\right)=\{\begin{array}{cc} E_{d}+\frac{E_{s}-E_{d}}{2}\{1-cos(\pi \frac{t}{T_{s}})\} & 0\leq t\leq T_{s}\\ E_{d}+\frac{E_{s}-E_{d}}{2}\{1+cos(2\pi \frac{t-T_{s}}{T_{s}})\} & T_{s}<t\leq \frac{3}{2}T_{s}\\ E_{d} & \frac{3}{2}T_{s}<t\leq T_{rr} \end{array} \end{array}$$ (68)

Note that, *E*_*d*_, *E*_*s*_ represents LV elastance at end-diastole and end-systole, respectively. $T_{rr},T_{s}\left(=0.3\times \sqrt{T_{rr}}\right)$ represents cardiac cycle length and duration of LV activation calculated by Bazett equation [46].

From the above equation, we can find that in the TVEM, *E*_*inst*_ and *E*_*load*_ always acquire the same value of *E*(*t*).

## Discussion

### Physical and mathematical interpretation of *E*_*inst*_ and *E*_*load*_

As explained in the Introduction, if we consider LV as an elastic compartment, the physical elastance of LV becomes *E*_*inst*_ which represents the transient ratio between the compartment pressure and the volume. The mathematical analysis of *E*_*inst*_ shown in section *E_inst_* in the simplified hemodynamic model revealed that *E*_*inst*_ is directly related with the cellular contraction force, affected by the LV radius by the effect of Laplace’s law. As the cellular contraction force is generated by *Ca*^2+^ concentration with certain delay from *Ca*^2+^, *E*_*inst*_ becomes almost constant during the ejection phase, as shown in Fig 9.

On the other hand, *E*_*load*_ is derived from the concept of the time varying elastance model which came from the concept of *E*_*max*_. The concept of the time varying elastance model is based on the assumption that *V*_*lv*_ at *P*_*lv*_ = 0 is always same, thus at the same cardiac phase time, the LV physical compartment elastance becomes the same, thus the pressure and the volume points lie on the same straight line. However, if we use the circulation model with detailed cellular contraction model, the pressure and the volume points lied on the line whose volume intercept move with time, which is different from the time varying elastance concept (Figs 6 and 7).

Mathematically, *E*_*load*_ is defined by Eq (26), which has similar representation with the analysis of the stability of the limit cycle in the bifurcation analysis. Thus, we can understand that *E*_*load*_ corresponds to the movement of the periodic limit cycle with changes in *R*_*out*_. And the difference between the values of *E*_*inst*_ and *E*_*load*_ can be understood from this consideration. The analysis of the slope of *E*_*load*_ in section *E_load_* in the simplified hemodynamic model clarified that the slope itself comes from the initial force velocity relation, where the initial velocity is determined by the rate of volume change related with *R*_*out*_. The latter analysis clarified that the slope is kept almost linear during the ejection phase, which finally results in the linear ESPVR.

From the above consideration, the index which directly represent the elastance of LV is *E*_*inst*_, however in the clinical situation, the important index of the LV function is the resulting hemodynamics which corresponds to the periodic limit cycle of the LV pressure and the volume. This may be the reason that *E*_*max*_ which is *E*_*load*_ at end-systole is accepted as the good index of LV contractility.

### Relation between *E*_*max*_ and *E*_*load*_

*E*_*max*_ is the slope of ESPVR proposed as an index of cardiac contractility [3, 47]. From the simulation results, the value of *E*_*load*_ at 280 [ms] after the onset time of the ejection, which was close to that of the end-systole, was close to *E*_*max*_. With our hemodynamic model, the end-systolic time slightly changed with respect to the afterload (*R*_*out*_); thus, the isochronous *F*_*ext*_—*L* relation at a fixed time close to the end-systole time became slightly different from the ESPVR. However, from the simulation results, the end-systolic time was almost linear with the LV end-systolic pressure (*P*_*lv*_); thus, the end-systolic points were in a line and the difference between the line and the isochrones of *F*_*ext*_—*L* was very small.

### Relation between force-velocity relation and *E*_*load*_

In our analysis, the initial isochronous *P*_*lv*_—*V*_*lv*_ relation slope was determined by the initial transient changes in the half-sarcomere length and cellular contraction force, as explained in section step 1. Moreover, the slope was approximately maintained during the ejection phase. In this period, the sarcomere shortening velocity was determined by the mechanical load to the cell corresponding to the force-velocity relation (FVR); thus, the slope, i.e., *E*_*load*_ was strongly related to the FVR characteristics but not to the cardiac tissue contractility.

Since the shape of FVR is known to be physiologically non-linear [48–50], the isochronous PV relation should become non-linear. However, as the range of the force variation is quite small, FVR becomes approximately linear, resulting in a linear isochronous PV relation.

On the other hand, the positive inotropic effect, such as the noradrenergic stimulation, increases *E*_*max*_, which corresponds to the increase in *E*_*load*_. This can be explained by the changes in FVR under the positive inotropic effect. As reported in Sonnenblick et al. [48–50], the positive inotropic effect changes the properties of FVR. In our model, we did not include the inotropic effect. Thus, this aspect must be considered in the future model.

### Time course of *E*_*inst*_ and *E*_*load*_

From Table 7, we can observe that *E*_*inst*_ slightly increases and then decreases, while *E*_*load*_ monotonically decreases during the ejection phase. As shown in section *E*_*inst*_ in the simplified hemodynamic model, *E*_*inst*_ directly reflects cellular contractility. Thus, the time course of *E*_*inst*_ is similar to the cellular contraction force (*F*_*b*_). Additionally, as shown in section *E*_*load*_ in the simplified hemodynamic model, *E*_*load*_ reflects the characteristics of FVR. Therefore, the time course of *E*_*load*_ is considered to reflect the time course of the ratio between $\frac{dF_{b}}{dt}$ and $\frac{dL}{dt}$.

### Comparison between the simplified hemodynamic model and TVEM

**Fig 17:**
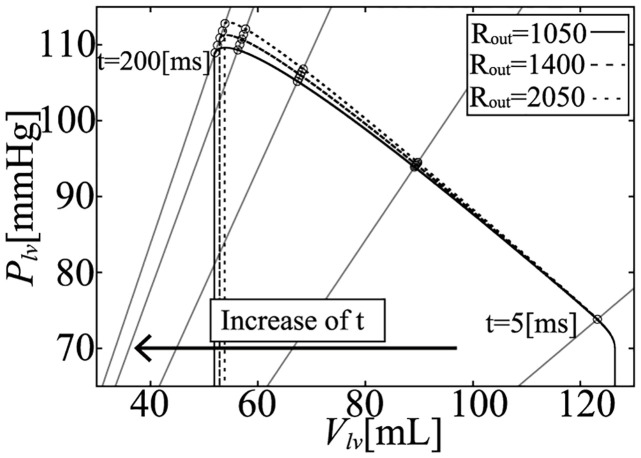
Isochronous *P*_*lv*_—*V*_*lv*_ relations during the ejection phase when using TVEM.

**Fig 18:**
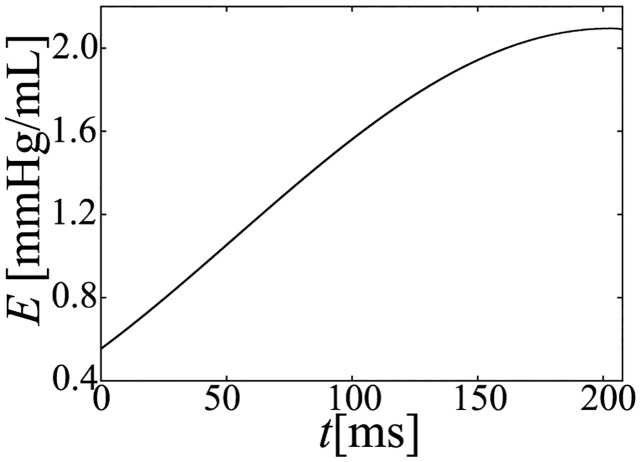
Time course of elastance (E) after the onset time of ejection when using TVEM.

Using a model in which the TVEM is used instead of the simplified hemodynamic model of the LV compartment, which was used by Heldt et al. [26], we evaluated the PV loops under the following peripheral resistance conditions: *R*_*out*_ = 1050, 1200, 1400, 1650, and 2050 [mmHg ⋅ ms/mL]. The resulting PV loops and isochronous of the *P*_*lv*_—*V*_*lv*_ relations at 5, 50, 100, 150, and 200[ms] after the onset time of ejection are shown in Fig 17. Moreover, the time course of elastance (*E*) is shown in Fig 18. As shown in Fig 17, the slope of the isochronous *P*_*lv*_—*V*_*lv*_ relation monotonically increases. In addition, as shown in Fig 18, *E* also increases monotonically. This is determined by the elastance function used for TVEM. As explained in section Simulation results, the time course of the *E*_*load*_ of our simplified hemodynamic model slightly decreases with time, which is different from that of the TVEM. Note that, the elastance function of TVEM varies with researchers, thus the time course of elastance changes with each research, however, since the function is constructed from the changes in the physiological pressure and volume, basic characteristics becomes similar.

For the animal experiment, Nishioka et al. [51] measured the isochronous PV relation in dogs and the results came close to our simulation model. That is, the slope of the isochronous PV relation did not change with time, but the volume intercept changed. However, in their experiment, pressure and volume at the onset time of ejection were not fixed. Thus, we cannot say that their results correspond to those of our simulation, but there are some similarities with our results.

### Influence of simplification of the hemodynamic model

To simplify the mathematical analysis, we simplified the hemodynamic model proposed by Utaki et al. [24, 25] for two points. First, LV wall thickness was fixed at a constant length, and second, the relation between the LV internal radius (*R*_*lv*_) and LV volume (*V*_*lv*_) was simplified to linear. However, these simplifications may reduce the accuracy of the model.

**Fig 19:**
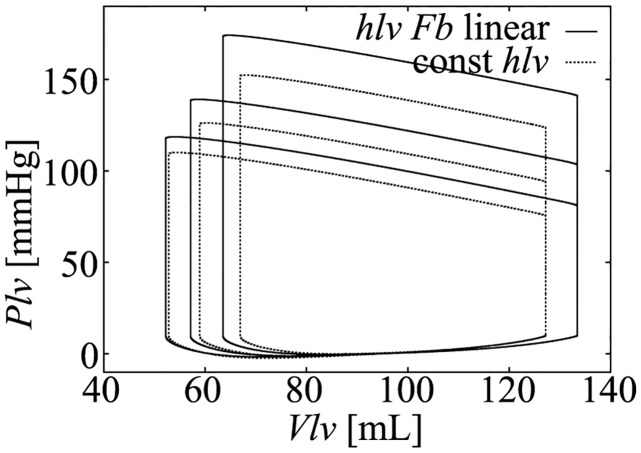
Simulation results using LV wall thickness linear with cellular contraction force *F*_*b*_ (hlv Fb linear) used in the original Utaki model [24], and fixed value (const hlv) used in the proposed simplified model. Note that, *R*_*out*_ = 1050, 1400, 2050 were used in the simulation.

Since the contribution of the LV wall thickning to the LV pressure is considered to be not small, the effect of fixing LV wall thickness (*h*_*lv*_) to the hemodynamics is not small. Simulation results of both models are shown in Fig 19, where the LV pressure becomes lower in the *h*_*lv*_ fixed model. However, the characteristics of the linear ESPVR is still preserved in the *h*_*lv*_ fixed model.

If we use the original *h*_*lv*_, which is proportional to the cellular contraction force (*F*_*b*_), the *E*_*inst*_ equation (Eq (27)) becomes as follows.

$$\begin{array}{c} E_{inst}=\frac{dP_{lv}}{dV_{lv}} \end{array}$$

$$\begin{array}{c} =\frac{\partial P_{lv}}{\partial R_{lv}}\frac{dR_{lv}}{dV_{lv}}+\frac{\partial P_{lv}}{\partial F_{ext}}\frac{dF_{ext}}{dV_{lv}}+\frac{\partial P_{lv}}{\partial h_{lv}}\frac{dh_{lv}}{dF_{b}}\frac{dF_{b}}{dV_{lv}} \end{array}$$ (69)

Comparing Eq (27) with Eq (69), we can see that the third term on the right-hand side of Eq (69) is ignored in our analysis. This term may lower the accuracy of the simulation results; however, from the simulation results shown in Fig 6, the simplified hemodynamic model still can reproduce physiological hemodynamics.

**Fig 20:**
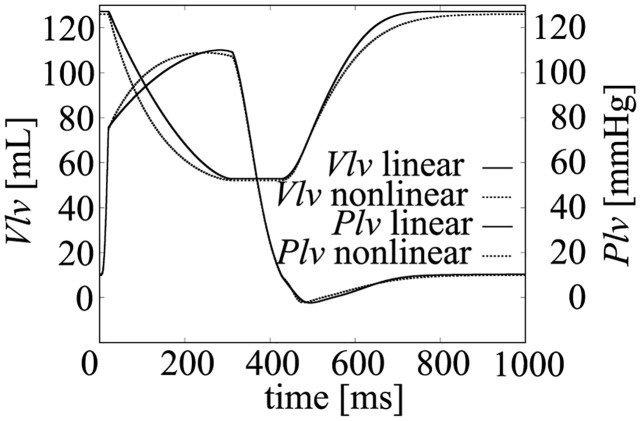
Simulation results using linear and nonlinear *R*_*lv*_ and *V*_*lv*_ relations.

As shown in Eq (2), the relation between the LV radius *R*_*lv*_ and the volume (*V*_*lv*_) is simplified to a linear relation in our model as shown in Fig 2. This simplification may affect the resulting pressure and volume time course. To validate that the simplification can reproduce physiological LV characteristics, the pressure and the volume time course with both the linear and the nonlinear relation were simulated in the condition without fixing *P*_*lv*_ and *V*_*lv*_ at the onset time of ejection (Fig 20). The results showed not small difference between these models, however, the time courses of the LV pressure and volume for both models are in the physiological range, so that the simulation results and the analysis results can be considered to be the results within the physiological conditions.

Additionally, as shown in Eq (54), the relation between the half-sarcomere length (*L*) and the LV volume (*V*_*lv*_) is simplified to a linear relation in our model, while the relation becomes non-linear in dogs, as reported by Rodriguez et al. [31]. In this case, the isochronous PV relation may become non-linear. In the case of rats and sheep, the isochronous PV relation is reported as non-linear [52, 53]. Thus, part of the difference with our simulation results may be caused by this simplification.

### Limitations of the analysis

In the analysis of step 2 (section step 2), the slope of the relation between the LV wall tension (*F*_*ext*_) and half-sarcomere length (*L*) is assumed to be fixed, while as shown in the simulation results (Table 7), the slope slightly decreases during the ejection phase. We could not derive an analytical solution without a fixed slope value; thus, in this aspect, we need further analysis of the mathematical equations of the model.

In the calculation of $\frac{dL}{dt}$ from Eq (56), $\frac{\partial V_{lv}}{\partial V_{tot}}$ and $\frac{\partial V_{lv}}{\partial F_{ext}}$ are calculated from Eqs (61) and (62). Note that, these equations cannot be calculated when $\frac{\partial P_{lv}}{\partial V_{lv}}=-\frac{1}{C_{a}}$.

### Relation with the 3D models

Computational modeling of cardiac function have now progressed to multi-scale modeling realizing 3D geometry coupled with cellular functions and circulating fluid dynamics [54–58], and reproducing pathological conditions [59].

These models are intended to use detailed elements in the whole model to reproduce accurate behavior of the heart, however, since the large deformation elastic model calculation often becomes unstable, the elements used in these models are often simplified to reproduce physiological results [60]. Thus the reproduced aortic pressure, flow velocity or ventricular motion of these models have fair similarities with the clinical data, however, detailed analysis on the relation between the hemodynamics and the microscopic molecular dynamics are often difficult, and also the mathematical analysis of the dynamics of these models are often impossible. On the other hand, as shown in the results, the simplified lumped models of LV and circulation system have disadvantages in reproduction accuracy, however, such models are still useful since they are capable of being mathematically analyzed for their dynamics.

## Conclusions

In this paper, we evaluated the simulation results of the proposed hemodynamic model that included a detailed cellular contraction model. By defining two different LV elastances: 1) instantaneous elastance and 2) load-dependent elastance, we were able to evaluate these elastances from the simulation model and found that these elastances showed markedly different time courses. That is, the instantaneous elastances showed a bell-shaped curve corresponding to the cellular contraction force, while the load-dependent elastance hardly changed with time. We then analyzed the mechanism that determines these elastances from the model equations, and found that the instantaneous elastance directly coincided with the cellular contraction force, while the load-dependent elastance was determined by the characteristics of both the instantaneous isovelocity-shortening and the force-velocity relation of cardiac cells. The slope of the isochronous pressure-volume relation was mainly determined by the velocity-dependent force drop characteristics in the instantaneous shortening. Moreover, the linear relation between the isochronous pressure and volume was based on the characteristics that the relation between the time derivative of the cellular contraction force and the cellular shortening velocity becomes linear, which comes from the combined characteristics of the LV and aortic compliances.

## Supporting information

S1 Model Source Code Contains C program code of the model. (ZIP)

S1 Model Equation Contains model equations. (PDF)
