# Supplementary material for: Mathematical analysis of left ventricular elastance with respect to afterload change during ejection phase
Source: PLoS Comput Biol. 2024 Apr 18;20(4):e1011974. doi: 10.1371/journal.pcbi.1011974 (PMC11025827; doi:10.1371/journal.pcbi.1011974)
Supplement: S1 Model Source Code — (ZIP) [file pcbi.1011974.s001.zip › circulation_Clangver/documentation/HowToRun.html]

HowToRun


 

# How to run simulation models

This document explains how to use this package.

## Folders and Contents

```
📂 circulation_Clangver/
  - 📂 designs/ : sequence diagrams for simulation programs
  - 📂 documentation/
    - "HowToRun.md" : this document
  - 📂 simulations/
    - 📂 bin/
      - "run_isovelocity_contraction.exe" : binary file for isovelcoity contraction experiment.
      - "run_simplified_hemodynamic_model.exe" : binary file for simplified hemodynamic model simulation
      - "run_tvem.exe" : binary file for time varying elastance model
    - 📂 build/
      - 📂 isovelocity_contraction/
        - "build.bat" :  compile script for windows
        - "build.sh" : compile script for linux
      - 📂 simplified_hemodynamic_model/
        - "build.bat" :  compile script for windows
        - "build.sh" : compile script for linux
      - 📂 tvem/
        - "build.bat" :  compile script for windows
        - "build.sh" : compile script for linux
    - 📂 configuration/
      - "isovelcoity_contraction_variables.ini" : configuration file for isovelcity contrction experiment
      - "simplified_hemodynamic_model_variables.ini" : configuration file for simplified hemodynamic model
      - "tvem_variables.ini" : configuration file for time varying elastance model
    - 📂 results/
      - 📂 isovelocity_contraction/ : the directory to place simulation result
      - 📂 simplified_hemodynamic_model/ : the directory to place simulation result
      - 📂 tvem/ : the direcotry to place simulation result
    - 📂 src/ : all source codes are here
      - 📂 calculation/ 
      - 📂 configuration_reader/
      - 📂 csvmaker/
      - 📂 logging/ 
      - 📂 main/ : has each main .c files
      - 📂 models/
        - 📂 circulation/ 
        - 📂 contraction/ 
        - 📂 left_ventricle/
```

## Environment Prerequisites

- OS

  - Windows10
  - Linux
- Compiler

  - GCC compiler

## Compile

- for windows

  1. move the directory named "circulation\_Clangver/simulations/build/*{model name you want to complile}*/" in file explore
  2. double-click the file named **"build.bat"**
  3. automatically opened command prompt, start compiling
  4. after compiling, run\_*{model name you built}*.exe is generated in the directory named "circulation\_Calngver/simulations/bin/"
- for linux

  1. move the directory "circulation\_Clangver/simulations/build/{model name you want to complile}/"
  2. execute the following command

  ```
  ./bash build.sh
  ```

  3. after compiling, run\_*{model name you built}*.exe is generated in the directory named "circulation\_Calngver/simulations/bin/"

## Program Usages

### Simplified Hemodynamic model

#### Configuring Simulation Conditions

Edit the variables in "circulation\_Clangver/simulations/configuration/simplified\_hemodynamic\_model\_variables.ini" and save it.  
The main simulation conditions are explained as followings.

| variable name | type | explanation |
| --- | --- | --- |
| fixPV\_tEJ | int(0 or 1) | flag to fix the aorta pressure and volume at onset time of ejection |
| Pa\_fixed | double | parameter to fix the aorta pressure at onset time of ejection |
| Rout | double | parameter to change afterload |
| Vlv\_inst\_decrease | int(0 or 1) | flag to instantaneously decrease left ventricular volume |
| Vlv\_inst\_decrease\_time | double | parameter to change Vlv\_inst\_decrease time. |
| Vlv\_inst\_decrease\_ratio | double | parameter to change Vlv\_inst\_decrease ratio(%). |
| radius\_volume\_linear\_relation | int(0 or 1) | flag to use linear L-Rlv relation |
| const\_wall\_thickness | int(0 or 1) | flag to use constant wall\_thickness |
| save\_interval | double | parameter to save. set time interval which you'd like to save. |
| save\_start\_time | double | parameter to determine saving start time. set start time which you'd like to save. |

#### Execution

1. Move to the directory (circulation\_Clangver/simulations/bin/).
2. double-click the .exe file "run\_simplified\_hemodynamic\_model.exe" (in case of linuxOS, execute command "./run\_simplified\_hemodynamic\_model.exe")
3. the simulation result is generated the directory(circulation\_Clangver/simulations/results/simplified\_hemodynamic\_model/) and it is named "{*YYYYDDDDHHMMss*}.csv".

### TVEM(Time Varying Elastance Model)

#### Configuring simulation conditions

Edit the variables in "circulation\_Clangver/simulations/configuration/tvem\_variables.ini" and save it.  
The main simulation conditions are explained as followings.

| variable name | type | explanation |
| --- | --- | --- |
| fixPV\_tEJ | int(0 or 1) | flag to fix the aorta pressure and volume at onset time of ejection |
| Pa\_fixed | double | parameter to fix the aorta pressure at onset time of ejection |
| Rout | double | parameter to change afterload |
| save\_interval | double | parameter to save. set time interval which you'd like to save. |
| save\_start\_time | double | parameter to determine saving start time. set start time which you'd like to save. |

#### Execution

1. Move to the directory (circulation\_Clangver/simulations/bin/).
2. double-click the .exe file "run\_tvem.exe" (in case of linuxOS, execute command "./run\_tvem.exe")
3. the simulation result is generated the directory(circulation\_Clangver/simulations/results/tvem) and it is named "{*YYYYDDDDHHMMss*}.csv".

### Isovelocity Contraction

#### Configuring simulation conditions

Edit the variables in "circulation\_Clangver/simulations/configuration/isovelocity\_contraction\_variables.ini" and save it.  
The main simulation conditions are explained as followings.

| variable name | type | explanation |
| --- | --- | --- |
| L | double | parameter to determine initial sarcomere length |
| dLdt | double | parameter to determine sarcomere shortening velcoity |
| t\_shortening | double | parameter to determine shortening start time |
| save\_interval | double | parameter to save. set time interval which you'd like to save. |
| save\_start\_time | double | parameter to determine saving start time. set start time which you'd like to save. |

#### Execution

1. Move to the directory (circulation\_Clangver/simulations/bin/).
2. double-click the .exe file "run\_isovelocity\_contraction.exe" (in case of linuxOS, execute command "./run\_isovelocity\_contraction.exe")
3. the simulation result is generated the directory(circulation\_Clangver/simulations/results/isovelocity\_contraction) and it is named "{*YYYYDDDDHHMMss*}.csv".
