# Supplementary figures and images for: Mathematical analysis of left ventricular elastance with respect to afterload change during ejection phase
Source: PLoS Comput Biol. 2024 Apr 18;20(4):e1011974. doi: 10.1371/journal.pcbi.1011974 (PMC11025827; doi:10.1371/journal.pcbi.1011974)

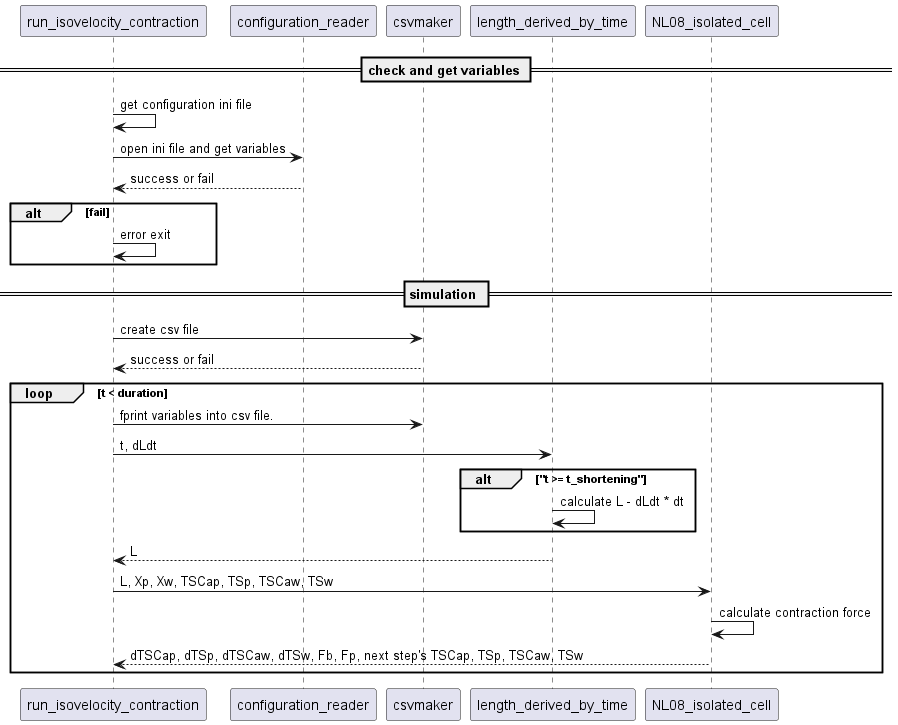

Supplement: S1 Model Source Code — (ZIP) [file pcbi.1011974.s001.zip › circulation_Clangver/designs/isovelocity_contraction.png]

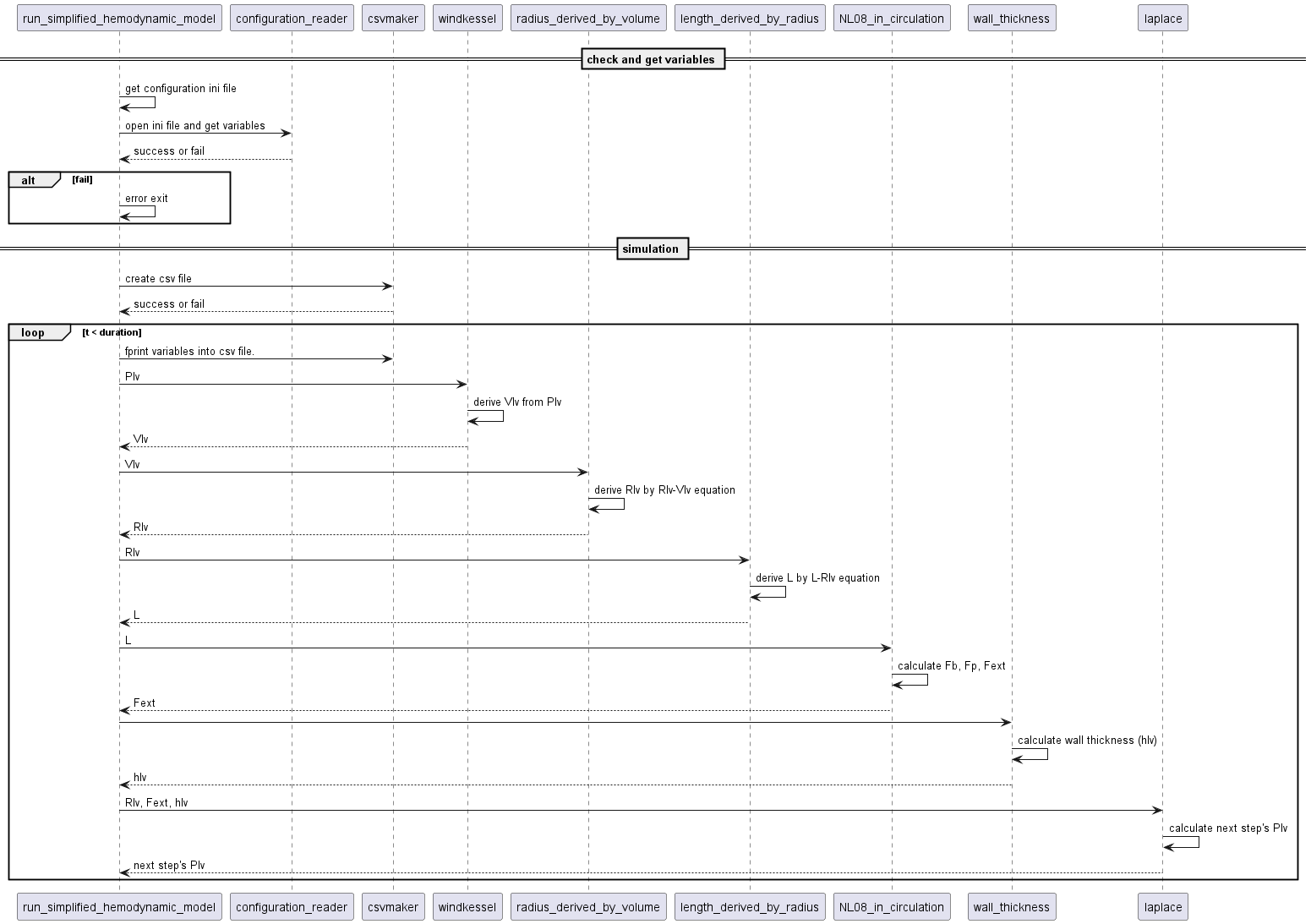

Supplement: S1 Model Source Code — (ZIP) [file pcbi.1011974.s001.zip › circulation_Clangver/designs/simplified_hemodynamic_model.png]

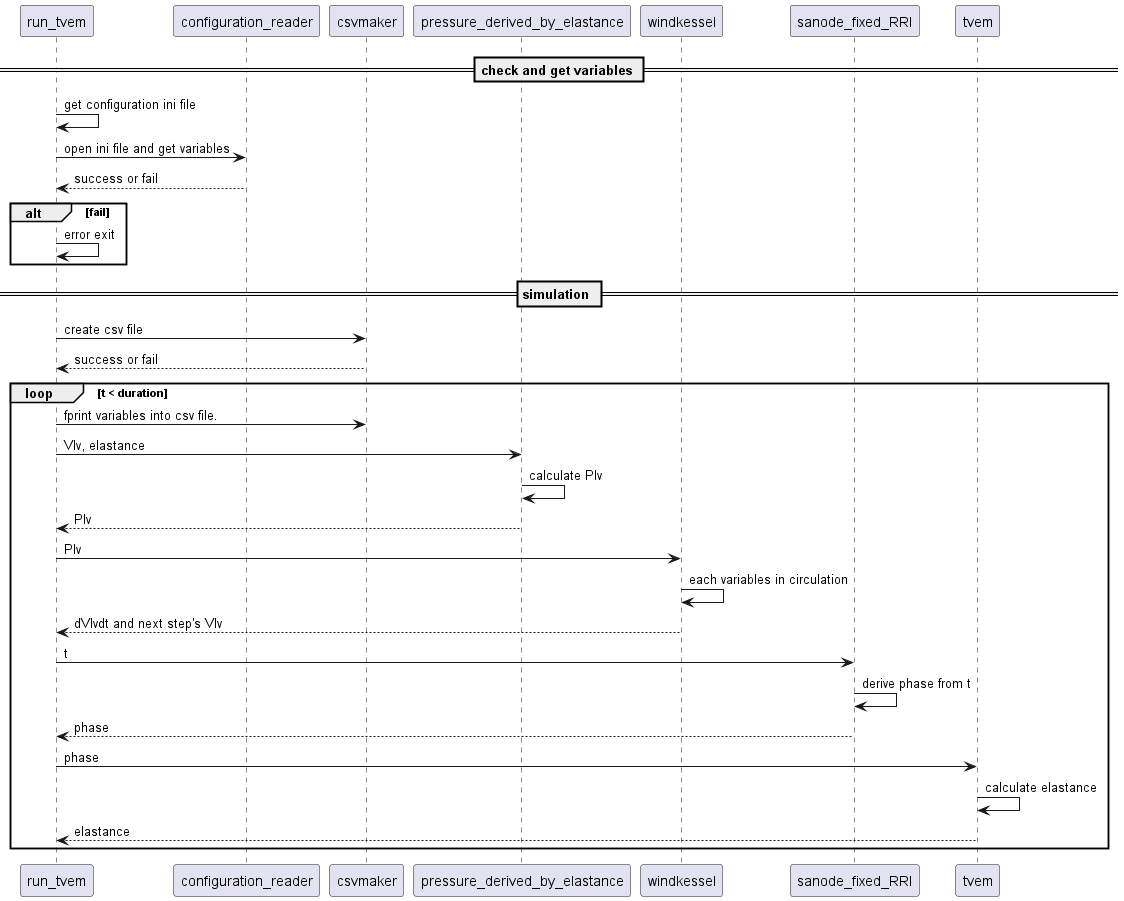

Supplement: S1 Model Source Code — (ZIP) [file pcbi.1011974.s001.zip › circulation_Clangver/designs/tvem.png]
