# Supplementary material for: Mathematical analysis of left ventricular elastance with respect to afterload change during ejection phase
Source: PLoS Comput Biol. 2024 Apr 18;20(4):e1011974. doi: 10.1371/journal.pcbi.1011974 (PMC11025827; doi:10.1371/journal.pcbi.1011974)
Supplement: S1 Model Equation — (PDF) [file pcbi.1011974.s002.pdf]

Supplementary Material

1 model equations

1.1 circulation model

$$q_0 = \begin{cases} (P_{E1} - P_{lv})/R_{pv} & P_{E1} > P_{lv} \\ 0 & otherwise \end{cases} \tag{1}$$

$$q_{in} = \begin{cases} (P_{lv} - P_a)/R_{lo} & P_{lv} > P_a \\ 0 & otherwise \end{cases} \tag{2}$$

$$q_{out} = (P_a - P_{E2})/R_{out} \tag{3}$$

$$P_a = V_a/C_a \tag{4}$$

$$\frac{dV_a}{dt} = q_{in} - q_{out} \tag{5}$$

$$\frac{dV_{lv}}{dt} = q_0 - q_{in} \tag{6}$$

1.2 LV geometry model

$$R_{lv} = K_R V_{lv} + K_V \tag{7}$$

$$L = K_L R_{lv} + L_b \tag{8}$$

$$h_{lv} = h_{lvED} \tag{9}$$

$$P_{lv} = \frac{2K_u F_{ext} h_{lv}}{R_{lv}} \tag{10}$$

1.3 mechanical part of the contraction model

$$h_w = L - X_w \tag{11}$$

$$h_p = L - X_p \tag{12}$$

$$\frac{dX_w}{dt} = B(h_w - h_{wr}) \tag{13}$$

$$\frac{dX_p}{dt} = B(h_p - h_{pr}) \tag{14}$$

$$F_{ext} = K_S F_b + F_p \tag{15}$$

$$F_p = \begin{cases} -K_{PL}\left(1 - \frac{L}{L_0}\right) & L < L_0 \\ K_{PE}\left(e^{D\left(\frac{L}{L_0}-1\right)} - 1\right) & otherwise \end{cases} \tag{16}$$

$$F_b = A_w([TSCa_3^{\sim}] + [TS^{\sim}])h_w + A_p([TSCa_3^{*}] + [TS^{*}])h_p \tag{17}$$

1.4 chemical part of the contraction model

$$\frac{d[TSCa_3]}{dt} = Y_b[TS][Ca^{2+}]^3 - Z_b[TSCa_3] + g[TSCa_3^{\sim}] - f[TSCa_3]_{\text{eff}} \tag{18}$$

$$\frac{d[TSCa_3^{\sim}]}{dt} = f[TSCa_3]_{\text{eff}} - g[TSCa_3^{\sim}] + Z_p[TSCa_3^{*}] - Y_p[TSCa_3^{\sim}] \tag{19}$$

$$\frac{d[TSCa_3^{*}]}{dt} = Y_p[TSCa_3^{\sim}] - Z_p[TSCa_3^{*}] + Z_r[TS^{*}][Ca^{2+}]^3 - Y_r[TSCa_3^{*}] \tag{20}$$

$$\frac{d[TS^{*}]}{dt} = Y_r[TSCa_3^{*}] - Z_r[TS^{*}][Ca^{2+}]^3 + Z_q[TS^{\sim}] - Y_q[TS^{*}] \tag{21}$$

$$\frac{d[TS^{\sim}]}{dt} = Y_q[TS^{*}] - Z_q[TS^{\sim}] - g_d[TS^{\sim}] \tag{22}$$

$$[TS] = [TS]_t - [TSCa_3] - [TSCa_3^{\sim}] - [TSCa_3^{*}] - [TS^{*}] - [TS^{\sim}] \tag{23}$$

$$[TSCa_3]_{\text{eff}} = e^{-R(L-L_a)^2}[TSCa_3] \tag{24}$$

$$g = Z_a + Y_v\left(1 - e^{-\gamma_m(h_w - h_{wr})^2}\right) \tag{25}$$

$$g_d = Y_d + Y_c(L-L_c)^2 + Y_{vd}\left(1 - e^{-\gamma_m(h_w - h_{wr})^2}\right) \tag{26}$$

$$\gamma_m = \begin{cases} \gamma\frac{1}{K_\gamma} & \frac{dX_w}{dt} > 0 \\ \gamma & otherwise \end{cases} \tag{27}$$

1.5 calcium dynamics of the contraction model

$$Q_{rel} = Q_m\left(\frac{t}{t_1}\right)^4 e^{4\left(1-\frac{t}{t_1}\right)} + Q_{pump\_rest} \tag{28}$$

$$Q_{pump} = K_p\frac{1}{1+\left(\frac{K_m}{[Ca^{2+}]}\right)^2} \tag{29}$$

$$\frac{d[Ca^{2+}]}{dt} = Q_{rel} - Q_{pump} - I_{troponin} \tag{30}$$

$$I_{troponin} = 3\left(\frac{d[TSCa_3]}{dt} + \frac{d[TSCa_3^{\sim}]}{dt} + \frac{d[TSCa_3^{*}]}{dt}\right) \tag{31}$$
